# Opioidergic modulation of monetary incentive delay fMRI responses

**DOI:** 10.1007/s00213-025-06753-7

**Published:** 2025-02-12

**Authors:** Samuel Turton, Peter C.T. Hawkins, Christopher Muller-Pollard, Evangelos Zois, Patricia Conrod, Fernando Zelaya, Mitul A. Mehta

**Affiliations:** 1 Institute of Psychiatry, Psychology and Neuroscience, King’s College London, London, UK; 2 Division of Psychiatry, Imperial College London, London, UK; 3 Neurodevelopmental Service Sussex Partnership NHS Foundation Trust, London, UK; 4 IU International University of Applied Sciences, Bad Honnef, Germany; 5 Department of Psychiatry, Université de Montreal, CHU Ste Justine Hospital, Montreal, QC Canada

## Abstract

**Rationale:**

It is hypothesised that modulation of striatal dopaminergic signalling plays a key role in the rewarding effects of opioids. The monetary incentive delay (MID) task is a functional magnetic resonance imaging (fMRI) paradigm used to investigate striatal responses, which may reflect striatal dopamine release, during the anticipation of a financial reward.

**Objectives:**

We hypothesised that fentanyl would modulate striatal MID task Blood Oxygenation Level Dependent (BOLD) responses, reflecting opioidergic modulation of striatal dopaminergic signalling.

**Methods:**

24 right-handed males who undertook four MRI scanning sessions, during which they completed an MID task 15 min after receiving an intravenous infusion of either one of two doses of fentanyl (50 µg/70kg), naloxone (400 µg) or placebo (saline 0.9%), were included in the analyses. End tidal CO_2_ data were collected to control for respiratory depression.

**Results:**

We demonstrated fentanyl induced increases in MID task reward and loss anticipation BOLD compared with placebo and naloxone in both region of interest (ROI) and whole brain analyses. These results were in cortical regions including the lingual gyrus, precuneus, posterior cingulate and frontal pole rather than the striatum.

**Conclusions:**

Our results show the primary effects of fentanyl on MID anticipation BOLD in regions associated with the preparation of a motor response to a salient visual cue, rather than in regions typically associated with reward processing such as the striatum. This suggests that opioid agonists do not affect striatal activation during the MID task. Tasks using naturalistic rewards, for example feeding, sex or social contact which induce endogenous opioid signalling, may be more appropriate to probe the effects of fentanyl on reward processing. These results are from male participants’ data and therefore may not be generalisable to female participants.

**Supplementary Information:**

The online version contains supplementary material available at 10.1007/s00213-025-06753-7.

## Introduction

The endogenous opioid system includes mu (MOR), kappa (KOR) and delta (DOR) receptors, and a range of endogenous ligands (Pathan and Williams 2012) and plays a significant role in reward processing, particularly the modulation of the hedonic value or ‘liking’ of a reward by MORs (Berridge and Kringelbach 2015). For example, MOR agonists directly administered to the ventral striatum, ventral pallidum or orbitofrontal cortex increase hedonic liking responses to rewards in rats (Berridge and Kringelbach 2015; Castro and Berridge 2017).

Endogenous opioids also modulate mesocorticolimbic dopaminergic neuron signalling via MORs on ventral tegmental area (VTA) GABA-ergic and glutamatergic interneurons (McGovern et al. 2023). These dopaminergic neurons project to regions rich in dopamine and mu-opioid receptors, including the ventral and dorsal striatum which are key in reward-related motivation, or ‘wanting’, and learning (Bromberg-Martin et al. 2010; Le Merrer et al. 2009; Malén et al. 2022).

Mesocorticolimbic dopamine signalling is important in the development and maintenance of opioid dependence (Koob and Volkow 2016) and MOR and dopaminergic signalling have close regional and functional associations in wanting and liking responses. However, these mechanisms are complex and regionally specific which adds significant complexity to understanding reward-related mechanisms when opioids are systemically administered, for example when smoked or intravenously (IV) injected. For example, sub-regional hedonic ‘hot-’ and ‘cold-spots’ exist in the ventral striatum where opioid agonists induce liking or disgust responses respectively (Berridge and Kringelbach 2015). Furthermore, MOR agonist induced feeding can be modulated by dopamine receptor antagonists, but this effect varies depending on whether the MOR agonists or dopamine antagonists are administered to the VTA or ventral striatum (MacDonald et al. 2004; Will et al. 2006). Additionally, there are extra-striatal brain regions that play a significant role in the interrelation between opioidergic and dopaminergic signalling, such as the periaqueductal grey matter, amygdala and hypothalamus (Reeves et al. 2022; Zhang et al. 2022). Given these factors, systemic opioids effects on reward processing must be considered on a ‘whole-brain’ level to better understand the mechanism of addiction to these drugs.

The monetary incentive delay (MID) task is a functional magnetic resonance imaging (fMRI) task commonly used to probe blood-oxygen-level-dependent (BOLD) responses during the anticipation, and following the receipt, of a monetary reward. The task consists of a cue indicating the type of trial to be performed which participants have previously been trained to associate with a reaction time dependent outcome (reward, neutral or loss), an anticipatory phase during which participants await a response target and an outcome or feedback phase detailing their performance for the trial (Wilson et al. 2018; Zeng et al. 2022). Anticipation and outcome phase activation overlap in the ventral and dorsal striatum and insula (Wilson et al. 2018; Zeng et al. 2022) reflecting the importance of these regions in both ‘wanting’ and ‘liking’ processes.

There is also evidence that striatal MID task reward anticipatory period BOLD responses may represent dopaminergic signalling. For example, reward anticipation BOLD responses have been shown to correlate with monetary reward task ventral striatal dopamine release, though the evidence of this is limited and inconsistent (Schott et al. 2008; Urban et al. 2012). Furthermore, striatal MID task reward anticipatory period BOLD responses can be modulated with dopamine agonists and antagonists (Hawkins et al. 2021; Knutson et al. 2004; Murphy et al. 2017), but again these finding are not consistent (Grimm et al. 2021).

Understanding the effect of systemic opioid receptor agonism on MID task BOLD responses may improve our understand of the effects of systemic opioids on wanting and liking responses in the human brain and also the interrelation between opioid and dopaminergic signalling in regions which play a key role in reward and addiction. This is not only relevant in opioid addiction, but also other drugs that activate endogenous opioid signalling such as alcohol and amphetamines (Colasanti et al. 2012; Mitchell et al. 2012).

There have been no published studies examining the effects of opioid agonists on the MID task brain responses, but a number of studies have examined the effects of opioid receptor antagonists showing mixed results. Naltrexone and nalmefene, non-specific opioid receptor antagonists, do not modulate reward anticipation BOLD in healthy controls, heavy drinkers or alcohol dependent individuals (Gowin et al. 2023; Nestor et al. 2017). However, when given in conjunction with an IV alcohol infusion nalmafene does lower reward anticipation BOLD responses (Quelch et al. 2017). Kappa opioid receptor specific JNJ-67,953,964 increases reward anticipation BOLD in anhedonic individuals, relative to placebo (Pizzagalli et al. 2020).

Here, we examined the impact of intravenously administered fentanyl, a selective mu-opioid receptor agonist (Stanley 1992) and naloxone, a non-selective opioid receptor antagonist (MOR, DOR and KOR) (Clark and Abi-Dargham 2019), on MID task fMRI measures in healthy volunteers. We have previously published results from the same experiments examining the effects of fentanyl on regional Cerebral Blood Flow (CBF) using Arterial Spin Labelling (ASL). This showed increased cerebral blood flow (CBF) in a number of regions related to the endogenous opiodergic system, including thalamus, lingual gyrus and para-hippocampal gyrus, following fentanyl administration (Zelaya et al. 2012). Fentanyl has a profound respiratory depressant effect (Dahan et al. 2005), but only cardiovascular effects at higher anaesthetic doses (Stanley and Webster 1978). The hypercapnia associated with opioid induced respiratory depression can induce changes in CBF and cerebral blood volume (CBV), both of which can have an impact on BOLD measures (Pattinson and Wise 2016). Therefore it is important to monitor for respiratory depressant effects of fentanyl during an fMRI scan.

Based on the evidence of the focus of pharmacological modulation of MID task reward anticipation responses, we hypothesised that IV fentanyl will increase striatal MID reward anticipation BOLD responses reflecting opioid induced increases in striatal dopaminergic signalling. Conversely, we hypothesised that IV naloxone will blunt striatal MID reward anticipation BOLD responses by reducing the effects of endogenous opioid signalling during the task.

We included a loss trial in the task to examine if the effects of opioid modulation are valence-specific. Previous research has shown salience and valence both have a modulatory role on MID reward anticipation BOLD contrast (Cooper and Knutson 2008), but a recent meta-analysis showed both reward and loss anticipation are associated with similar activation in the striatum, anterior insular and anterior cingulate (Wilson et al. 2018), so we tested the same regions for the effects of reward and loss trials..

We also carried out exploratory analyses to examine the effects of IV fentanyl and naloxone on other MID anticipatory and outcome contrasts.

## Methods

Our participants and scanning methods have been previously published (Zelaya et al. 2012) but are outlined below. More detailed methods are presented in the supplementary methods section.

27 right-handed male participants with no significant psychiatric, neurological or medical history were scanned for this study. All participants provided written consent to participate and the study was approved by Guy’s and St. Thomas’ Hospital Research Ethics Committee (06/Q0704/24).

Right handed males were chosen to reduce variance due to effects of brain lateralisation as well as gender driven influences, for example menstrual cycle. This, however, poses a potential limitation in generalising any results to a whole population including females.

Three participants were excluded from the analyses; 1 participant completed only 1 scan, 1 participant was excluded due to problems with spatial co-registration of the BOLD fMRI data and 1 participant was excluded due to excessive motion during all scans sessions. The remaining 24 participants (mean age 25.5 years, range 19 to 36 years) were included in the analyses. However, some individual scans were either missing (e.g. due to acquisition errors) or excluded due to excessive motion (see *Pre-Processing* section for details). Furthermore, some scans were excluded from certain contrasts, for example due to non-responses to neutral cues or not enough misses for a win vs. lose feedback contrasts. The total number of scans available for each contrast is detailed in Table S1.

### Study and scanning procedures

Participants underwent four MRI scanning sessions at least 1 week apart. During each session they received an intravenous infusion of one of the following: fentanyl (50 µg/70 kg) given on two sessions, naloxone (400 µg) or placebo (0.9% saline) in a randomised, double-blind design. Two doses of fentanyl were originally included in the design of the study carry out a test-retest analysis of fentanyl’s effects on our fMRI outcome measures.

15 min after the infusion participants completed an MID task consisting of high- and low-reward and loss cues (±£2.00 and ±£0.20 respectively) and a neutral cue. Participants completed subjective ratings of drug effects using a visual analogue scale (VAS) and end tidal CO_2_ (EtCO_2_) and respiratory rate data were collected. Further details of the MID task, study and scanning procedures are available in the Supplementary Methods section.

### Data analysis

Image pre-processing, modelling and analysis were all conducted in SPM 12 and used an established pipeline modified for this dataset (Hawkins et al. 2021) including slice time correction, two pass realignment, within subject co-registration to a T1-weighted image and spatial normalisation to MNI space using DARTEL. First and second level fMRI modelling were also carried out in SPM. Second level analyses used a random effects model and included F-Tests and post-hoc paired t-tests. Whole brain analyses used a threshold cluster level of *p* < 0.05 FWE and a cluster-forming threshold of *p* < 0.001. Full details are available in the supplementary methods section.

Due to issues with motion and task performance some participant’s individual scans were excluded from the analysis and the number of participants included in each contrast is available in Table S1. For a priori reward and loss anticipation contrasts: Fent1 vs. Fent2 *n* = 20, Fent1 vs. Plac *n* = 20, Fent1 vs. Nalox *n* = 17, Fent2 vs. Plac *n* = 21, Fent2 vs. Nalox *n* = 17, Plac vs. Nalox *n* = 17, Combined-Fent vs. Plac *n* = 21, Combined-Fent vs. Nalox *n* = 17.

Our two a priori anticipation period contrasts of interest were defined as combined high- & low-reward > neutral (herein referred to as reward anticipation) and combined high- & low-loss > neutral (loss anticipation), as we were not primarily interested in the differential effect of fentanyl or naloxone on the value of different rewards or losses. We also explored further anticipation and outcome contrasts as detailed in the supplementary methods. Whole brain analyses allowed us to combine fentanyl-1 and fentanyl-2 as a single contrast to improve our power examining fentanyl compared with placebo or naloxone. This was not possible with ROI analyses and therefore the effects of fentanyl-1 and fentanyl-2 were examined separately in ROI analyses. We also examined the separate effects of fentanyl-1 and fentanyl-2 in additional whole-brain analyses.

Five bilateral a priori regions of interest (ROIs) were used to explore the effects of fentanyl and naloxone on MID task BOLD responses: ventral striatum, caudate, putamen, anterior cingulate and anterior insula. These regions were chosen as they have significant MID task reward and loss anticipation BOLD contrast (Wilson et al. 2018) and high MOR density (Kantonen et al. 2020). Details of ROI definition are in the supplementary methods. ROI mean beta estimates for each contrast (see above) were extracted from using the SPM12 MarsBar.

Analysis of behavioural, VAS and ROI data were carried out in R (version 4.2.1). ROI data were analysed using linear mixed effects model to test for a within subject effect of ‘Drug’ and significant drug effects were then examined further with post-hoc paired t-tests. This is further detailed in the supplementary methods section.

### End-tidal CO_2_ (EtCO_2_)

Due to the potential effects of fentanyl induced respiratory depression and hypercapnia on BOLD signal we recorded EtCO_2_ for all participants in all scans. EtCO_2_ is a surrogate measure for circulating blood CO_2_ concentrations. Possible methods to maintain a constant EtCO_2_ and therefore minimise the effects of respiratory depression on BOLD signal include active adjustment of participants’ inhaled gas mixtures or mechanical ventilation (Pattinson and Wise 2016). However, these methods would add significant complexity to the study and therefore were not used.

EtCO_2_ measurements were recorded at a rate of one per second using nasal canula attached to a gas analyser (AEI Technologies, PA) and logged using inhouse software. This was used as a representative measure of arterial partial pressures of CO_2_. The archived EtCO_2_ data used in our analyses were available at 5 s intervals. We used the collected EtCO_2_ data to assess if our doses of fentanyl had a significant respiratory depressant impact. We also added ∆EtCO_2_ as a nuisance covariate to the ROI linear mixed effects model in R and the whole-brain SPM models to explore the potential confounding effect of changes in EtCO_2_ in all a priori and exploratory anticipation and outcome contrasts. ∆EtCO_2_ was the proportional difference in mean EtCO_2_ during the entire MID task compared with mean EtCO_2_ prior to the drug infusion for each scanning session.

Due to difficulties with the capnograph, only 14 participants with full EtCO_2_ data sets (i.e. in all 4 scans) were available. Missing data was due to either no capnography data collected for an entire scan or missing capnography data for a significant portion of the scan, for example the entire pre-infusion period. Data were also excluded due to erroneous values for the entirety or significant portions of the scan period, for example EtCO_2_ recordings below 20 mmHg for the entire data collection period, reflecting errors in the capnography recording.

The primary analyses presented in this manuscript do not use EtCO_2_ as a covariate. However, due to the potential confounding effects of fentanyl induced respiratory depression and hypercapnia on our BOLD measures we carried out secondary analyses examining these effects. For these secondary analyses ∆EtCO_2_ was added as a covariate to the linear mixed effects models in R and the SPM second level model in our ROI and whole-brain analyses respectively and results are presented in a separate section below.

### Mu-opioid receptor PET maps

Regional [^11^C]carfentanil BP_ND_ values were extracted from a published PET atlas in 204 heathy individuals (https://neurovault.org/collections/GCELSAIA) (Kantonen et al. 2020).

These [^11^C]carfentanil BP_ND_ values were used to compare the relative mu opioid receptor density in regions or volumes with significant MID task BOLD contrast and examine regional correlations between [^11^C]carfentanil BP_ND_ and our a priori fentanyl > placebo win and loss anticipation BOLD contrasts from the whole brain analyses. Further details of these methods are available in the supplementary materials.

## Results

### Behavioural and subjective effects

For task accuracy, cue reaction time and total winnings there was no significant effect of drug (ANOVA *p* > 0.05, Table S2).

**Fig. 1:**
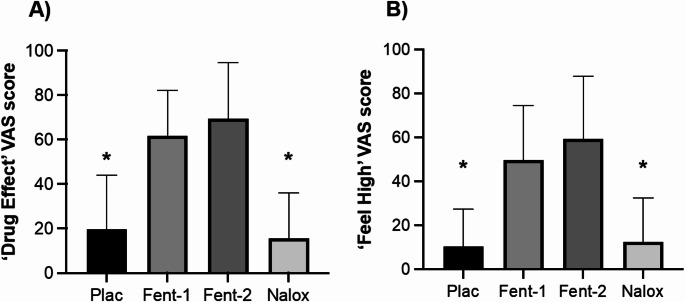
Visual analogue scale (VAS) rating of subjective effects of study medication immediately prior to starting MID task. (**A**) ‘drug effect’ and (**B**) ‘feel high’. * significant paired t-test (all *p* < 0.001) compared with both fentanyl-1 and fentanyl-2

There was a significant effect of drug on ‘drug effect’ and ‘feeling high’ VAS scales (both ANOVA *p* < 0.001). ‘Drug effect’ and ‘feeling high’ VAS rating for fentanyl-1 and fentanyl-2 was significantly higher than placebo and naloxone (all paired t-test *p* < 0.001), but there were no significant differences in scores between fentanyl-1 and fentanyl-2 or between placebo and naloxone (Fig. 1).

### BOLD-fMRI image analysis

There was no significant drug effect on head movement (total interframe motion in mm, ANOVA *p* > 0.05, Table S3).

**Fig. 2:**
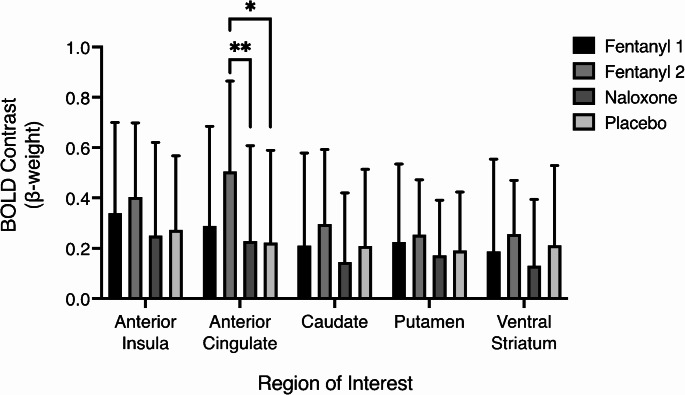
Mean (± SD) reward anticipation BOLD contrast (β-weight) across 5 a-priori ROIs for each drug condition. * paired-test *p* = 0.002. ** paired-test *p* = 0.009

A priori defined ROI analyses showed significant main-effect of Drug for both reward (F[3,341.7] = 6.2, *p* < 0.001) and loss anticipation BOLD contrasts (F[3,342.0] = 6.5, *p* < 0.001). Post-hoc paired tests showed fentanyl-2 had significantly greater reward anticipation BOLD contrast compared with placebo (*p* = 0.002) and naloxone (*p* = 0.009) (Fig. 2) and greater loss anticipation BOLD contrast compared with naloxone (*p* = 0.011) in the anterior cingulate ROI.

Exploratory mixed-model analyses showed a number of anticipation and feedback contrasts with a significant within-subject effect of drug (Bonferroni corrected *p* < 0.005, Table S4). Post-hoc paired tests showed one significant result after Bonferroni correction *p* < 0.002 within each contrast: *low-reward > neutral* anticipation fentanyl-2 > placebo (*p* < 0.002). There were no significant differences between naloxone and placebo or between fentanyl-1 and fentanyl-2 in any ROI across all contrasts.

### Whole brain analyses

Whole brain analyses examining the main effect of task (i.e. task based activation) for reward and loss anticipation contrasts for the placebo scan showed bilateral clusters of statistically significant difference in BOLD response in a number of expected regions including striatum, pallidum, thalamus, anterior insula and cingulate and motor cortices (Figure S1).

We did not observe any significant *fentanyl-1 > fentanyl-2* or *fentanyl-2 > fentanyl-1* clusters for any contrast.

**Fig. 3:**
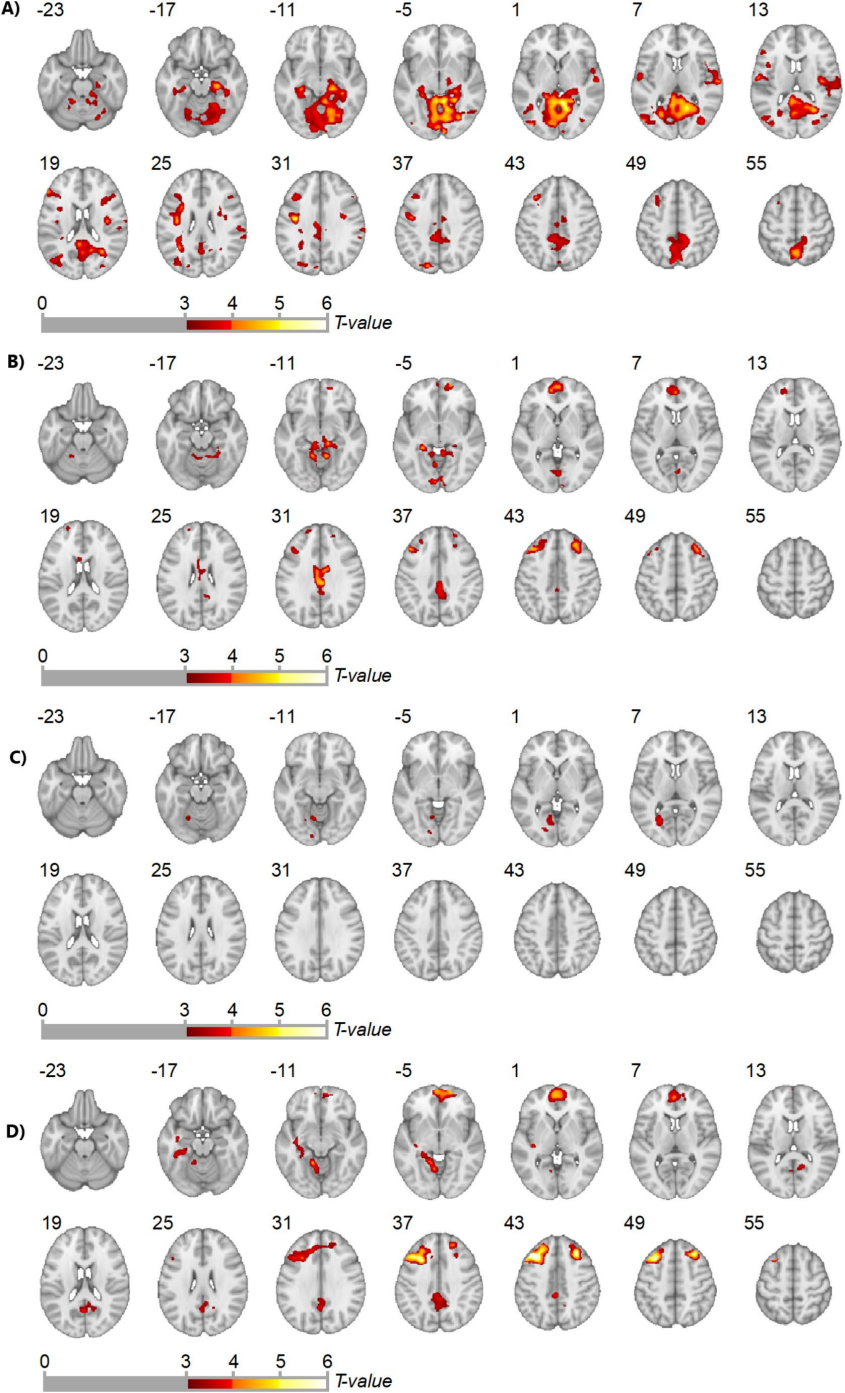
Paired test whole brain analysis results examining effect of drug on reward and loss anticipation contrasts (cluster level p-FWE < 0.05). Includes MNI z-coordinate and presented in neurological orientation (i.e. image left is subject’s left). (**A**) reward anticipation fentanyl > placebo. (**B**) reward anticipation fentanyl > naloxone. (**C**) loss anticipation fentanyl > placebo. (**D**) loss anticipation fentanyl > naloxone

A priori defined analyses examined the effect of fentanyl (i.e. combined fentanyl-1 and fentanyl-2) on reward and loss anticipation contrasts. There were significant ANOVA drug induced differences in activation (cluster-level p-FWE < 0.05) for both of these contrasts. This was explored further with paired tests which showed significant clusters for *fentanyl > naloxone* and *fentanyl > placebo* in both reward and loss anticipation contrasts (Fig. 3, Table S5).

There was a degree of overlap in *fentanyl > placebo* and *fentanyl > naloxone* clusters across the reward and loss anticipation contrasts. The significant *fentanyl > placebo* clusters were more posterior including the lingual gyrus, parahippocampal and posterior cingulate gyri, bilateral hippocampus, precuneus and opercular cortex. The significant *fentanyl > naloxone* clusters included more anterior regions such as anterior cingulate and paracingulate gyri and frontal pole (Fig. 3, Table S5).

Exploratory whole-brain analyses examining the effect of fentanyl in other anticipation and feedback contrasts showed significant clusters from the ANOVA analysis for *fentanyl > placebo* and *fentanyl > naloxone* for *high-reward > neutral* and *low-reward > neutral* anticipation contrasts and *fentanyl > naloxone* only for *high-loss > neutral* and *low-loss > neutral* anticipation contrasts (Table S6). These clusters had similar distribution to the *fentanyl > placebo* and *fentanyl > naloxone* clusters in our a priori analyses above with no significant clusters in striatal regions. There were no significant *placebo > naloxone* or *naloxone > placebo* results in any anticipation contrasts. There were no significant results for any feedback contrasts.

The separate effects of fentanyl-1 and fentanyl-2 compared with placebo and naloxone showed significant results for both fentanyl-1 and fentanyl-2 (Table S7), including reward anticipation fentanyl-1 > placebo and fentanyl-2 > placebo and loss anticipation fentanyl-1 > naloxone and fentanyl-2 > naloxone clusters.

### Correlations with subjective effects

We examined whole brain correlations between VAS scores of “feeling high” and “drug effect” 10 min following IV fentanyl administration and our a priori contrasts. We did not find any significant results (all FWE *p* > 0.05) for correlations between fentanyl-1, fentanyl-2 or combined-fentanyl and VAS scores for either a priori contrast.

### EtCO2

EtCO2 data were available for *n* = 14 participants. There was a significant effect of drug on mean MID task EtCO_2_ (ANOVA *p* = 0.044), ∆EtCO_2_ (ANOVA *p* < 0.001) and respiratory rate (ANOVA *p* = 0.007). Paired t-tests showed higher EtCO_2_ and lower respiratory rates following fentanyl compared with naloxone and placebo (Table S8).

Our a-priori linear mixed-model ROI analyses were re-run including ∆EtCO_2_ as a covariate. There remained a significant effect of drug for both reward (F[3,300.9] = 3.4, *p* = 0.018) and loss (f[3,308.7] = 5.7, *p* < 0.001) anticipation contrasts. There was no significant effect of ∆EtCO_2_ in either of the mixed-models (*p* = 0.936 and *p* = 0.347).

Adding ∆EtCO_2_ as a covariate to a priori whole-brain analyses reduced the spatial extent of significant clusters for reward anticipation *fentanyl > placebo* (1 cluster, cluster-level p-FWE 0.007) and loss anticipation *fentanyl > naloxone* (2 clusters, cluster-level p-FWE 0.035 and 0.031) contrasts, compared with our results above (Figure S2). The remaining significant a priori whole brain results above were no longer significant with ∆EtCO2 added as a covariate to the model.

To examine if these changes in our results were solely due to the addition of ∆EtCO2 to the model, or due to a reduction of participant numbers included in the EtCO2 models we re-ran the models without ∆EtCO2 but including only the 14 participants with available ∆EtCO2. This demonstrated that the model with 14 participants had diminished cluster sizes compared with our all participant models, but that addition of ∆EtCO2 to the model further diminished the size of significant clusters for the reward anticipation *fentanyl > placebo* BOLD contrast (Figure S2). The addition of ∆EtCO2 to the model minimally reduced the cluster size for loss anticipation *fentanyl > naloxone* BOLD contrast (Figure S2).

### Mu-opioid receptor PET maps

Mean [^11^C]carfentanil BP_ND_ values in our significant clusters ranged from 0.15 to 1.16 (Table S9), and included regions with higher MOR density, such as paracingulate and anterior cingulate gyrus, and regions with negligible MOR density, such as lingual gyrus and areas of the occipital cortex.

Overall, the majority of the regions (19/29) and clusters (7/13) where we saw significant results had [^11^C]carfentanil BP_ND_ values below the median regional value (0.86– Table S9).

There were no regional correlations between [^11^C]carfentanil BP_ND_ and a priori fentanyl > placebo reward or loss anticipation (Pearson’s correlation coefficient *p* = 0.640 and *p* = 0.489 respectively) corrected for spatial autocorrelation using BrainSMASH (Burt et al. 2020).

## Discussion

We found IV fentanyl increased BOLD responses during both reward and loss anticipation in the MID task. However, these findings were primarily in cortical areas rather in the subcortical regions which are more typically associated with MID task anticipation BOLD responses. We did not find any effect of fentanyl on outcome responses, nor any effect of naloxone on supressing anticipation or outcome responses.

### Fentanyl increases reward and loss anticipation BOLD responses

Our hypothesis that fentanyl would increase striatal MID task reward and loss anticipation BOLD responses was based on evidence that MORs in the VTA modulate mesocorticolimbic dopaminergic signalling which has a key role in incentive motivation, or wanting (Bromberg-Martin et al. 2010; Le Merrer et al. 2009; Malén et al. 2022; McGovern et al. 2023). Drug induced ventral striatal dopamine release has been hypothesised to be a key mechanism in the development of addiction (Volkow et al. 2019) but we did not observe fentanyl induced increases in ventral striatal signalling that might reflect this. However, the evidence that monetary reward anticipation BOLD signal might be directly correlated with striatal dopamine release is mixed (Schott et al. 2008; Urban et al. 2012) which complicates this interpretation of our results.

More broadly the human evidence of opioid induced striatal dopamine release is limited (Milella et al. 2023; Nutt et al. 2015) and our results suggest that the primary effect of MOR agonism during the MID task is not in high dopamine receptor density striatal regions (Malén et al. 2022), typically associated with reward related wanting or liking processing. Rather our results are in regions responsible for visual and spatial processing (e.g. lingual and parahippocampal gyri, precuneus and lateral occipital cortex), memory (e.g. hippocampus, parahippocampal gyrus and posterior cingulate), sensory-motor function (e.g. precentral gyrus), task directed behaviour (frontal pole) and attention (e.g. precuneus, posterior cingulate and middle frontal gyrus) (Aminoff et al. 2013; Cavanna and Trimble 2006; Grill-Spector et al. 2001; Koechlin 2011; Leech and Sharp 2014; Lemon 2008; Palejwala et al. 2021; Stevens et al. 2011). These functions are important during the anticipation period of the MID task, where a subject processes the visual cue and prepares a motor response to the target and there is evidence that these regions are active during MID reward anticipation (Wilson et al. 2018) and our results suggest that MOR agonism modulates these processes. This may be due to compensatory increased processing in these regions required for participants to maintain their performance due to the sedative or other effects of fentanyl. This would reflect prior evidence of the effects of opioids to impair cognition, particularly at higher doses (Van Steenbergen et al. 2019).

Another possibility is that MOR agonism plays a role in the interaction between attentional and response processing in these regions. Previous research has shown MOR agonism with morphine increases learning preference towards faster motor responses to gain a higher financial reward, whilst MOR antagonism with naltrexone reduces this learning (Eikemo et al. 2017). However, this study was not carried out in conjunction with neuroimaging to help understand which regional may be involved in these learning effects.

We have previously shown increased CBF in the para-hippocampal and lingual gyri following IV fentanyl in the same participants (Zelaya et al. 2012). This may also indicate that our MID task anticipation BOLD results reflect a broader physiological change in these cortical regions following IV fentanyl, rather than a specific effect of MOR agonism on salient reward or loss anticipation. It is not possible to assess if there are similar changes in CBF in other cortical regions we observed significant BOLD results in, such as precuneus, posterior and anterior cingulate, as there were limited number of slices in the ASL imaging high MOR density subcortical structures, such as the striatum and thalamus, was prioritised and therefore most cortical areas were not included in the limited coverage.

Fentanyl was associated with higher reward and loss anticipation BOLD contrast compared with both placebo and naloxone. Our results show similar regional distribution of fentanyl effects on both reward and loss anticipation, and we observed no effect of fentanyl on *high-reward > low-reward* and *high-loss > low-loss* contrasts. This suggests that fentanyl does not modulate brain responses related to the value of the monetary reward (i.e. valence), but rather has a general effect on the preparatory brain processes prior to responding to a ‘salient’ cue (Cooper and Knutson 2008).

Whilst there was a degree of overlap between *fentanyl > placebo* and *fentanyl > naloxone* anticipation BOLD contrast, the *fentanyl > placebo* contrast was more posterior (e.g. lingual gyrus, precuneus and posterior cingulate cortex) and the *fentanyl > naloxone* contrast was more anterior (e.g. frontal pole, anterior cingulate, paracingulate gyrus and frontal gyrus) across both reward and loss anticipation. Further exploratory analyses examining other anticipation period contrasts also showed similar posterior and anterior regional distribution of *fentanyl > placebo* and *fentanyl > naloxone* results.

It is unclear why there were regional differences between *fentanyl > naloxone* and *fentanyl > placebo* contrasts. It could reflect slightly different numbers of participants in each of these contrasts (*n* = 21 and *n* = 17 respectively). It is also possible that there is a non-specific ‘clamping’ of endogenous opioid signalling (MOR, KOR and DOR) during the task with naloxone (Clark and Abi-Dargham 2019), compared with placebo which results in a differential regional distribution. However, we did not find any significant difference in anticipation BOLD contrast compared between placebo and naloxone to support this as discussed below.

In our ROI analyses we observed that the second fentanyl infusion scan (fentanyl-2) was associated with significant increases in reward anticipation BOLD contrast in the anterior cingulate, but the first fentanyl infusion scan (fentanyl-1) was not. This may represent an order effect due to priming or conditioning of the participants following their first fentanyl infusion (Le Merrer et al. 2009). However, we did not observe any significant differences between the 1st and 2nd fentanyl scan BOLD responses in either ROI or whole brain analyses in any of the a priori or exploratory contrasts, which makes these findings difficult to interpret. Whilst two doses of fentanyl were included to carry out a test-retest analysis, our study was not designed to be powered for this analysis of the MID task, and may well be underpowered to adequately detect differences in fMRI measures between the first and second fentanyl scans.

### Mu-opioid receptor density and effects of fentanyl on MID anticipation responses

Regions with significant MID reward > neutral anticipation BOLD from a seed based d-mapping meta-analysis (Wilson et al. 2018) are amongst those with the highest MOR density (e.g. ventral striatum, thalamus, putamen, caudate, amygdala, anterior cingulate and insula). Our significant results from a priori whole-brain reward and loss anticipation contrasts cover areas with a range of MOR density, as demonstrated in our results extracting [^11^C]carfentanil BP_ND_ values (Table S9). These significant clusters include regions with higher [^11^C]carfentanil binding (e.g. paracingulate gyrus, anterior cingulate and insula), but most are within the lower half of regional [^11^C]carfentanil BP_ND_ values, and some regions such as the lingual gyrus have negligible MOR density. Furthermore, we did not observe significant results in ROI or whole brain analyses in the majority of the highest MOR density regions, including striatal regions, even though we hypothesised these would be where the main effects on fentanyl in the MID task would be observed. Similarly to our MID task results we observed effects of fentanyl in regions with higher and lower MOR density in our CBF analysis (Zelaya et al. 2012). We also found no significant correlations between regional MOR density and the effects of fentanyl on reward or loss anticipation in our data.

This suggests that our observed results are unlikely to be due to the direct local effects of MOR agonism by fentanyl, an instead represent indirect or ‘downstream’ effects of MOR agonism. This may reflect previous results that MOR availability in high binding regions is significantly correlated with BOLD contrast in distant lower binding regions during other behavioural tasks (Karjalainen et al. 2019; Sun et al. 2022).

### MID task outcome responses

We did not find any effect of fentanyl on our exploratory MID outcome contrasts. There is a degree of overlap in the regions showing significant activation during the MID task outcome and anticipation phases, including the ventral striatum, thalamus, orbitofrontal and ventromedial pre-frontal cortex (Oldham et al. 2018; Wilson et al. 2018), and these regions have high MOR density (Table S9).

Given the evidence that MORs modulate the hedonic value or liking of a reward during the consummatory period (Berridge and Kringelbach 2015), a significant impact of fentanyl on brain responses following the receipt of the monetary reward was expected.

Rather than reflecting reward consummatory activation, the outcome phase of the MID task may represent a prediction error signal (Oldham et al. 2018), and components of it may also represent a ‘spillover’ from the anticipatory phase BOLD signal rather than consummation of a reward (Demidenko et al. 2021). Exploring the effect of fentanyl on outcomes to naturalistic rewards which can be consumed within the scanner, for example palatable food, social or sexual stimuli (Eikemo et al. 2016; Massaccesi et al. 2024; Rabiner et al. 2011; Sescousse et al. 2013), may be more relevant for examining the effects of MOR modulation, as discussed below in relation to the lack of effect of naloxone in our task.

### Endogenous opioid signalling during MID task (i.e. effects of naloxone)

We did not observe any significant impact of naloxone on MID anticipation or outcome contrasts, compared with placebo. This replicated previous findings that opioid receptor antagonists naltrexone and naloxone do not modulate MID reward anticipation BOLD contrast (Gowin et al. 2023; Nestor et al. 2017). A lack of effect of opioid receptor blockade on MID anticipation BOLD responses may indicate that the monetary reward is not sufficient to activate phasic endogenous opioid signalling during the MID task. This could also be reflected in the lack of associations between MID reward anticipation responses and MOR density shown in healthy controls, antipsychotic treated individuals with schizophrenia and individuals with alcohol dependence or gambling disorder (Shatalina et al. 2023; Turton et al. 2024).

Stimuli known to induce endogenous opioid signalling, such as alcohol consumption (Mitchell et al. 2012) or naturalistic rewards such as feeding (Tuulari et al. 2017), might show attenuation of anticipation or reward outcome responses following opioid receptor blockade. For example, nalmefene blunts MID task reward anticipation BOLD following an IV alcohol infusion (Quelch et al. 2017) and naltrexone blunts erotic image BOLD reward responses more than financial reward responses (Buchel et al., 2018). Another study found MOR selective inverse agonist GSK1521498 blunted striatal BOLD responses during palatable food delivery, although naltrexone did not (Rabiner et al. 2011). Opioid receptor antagonists with high affinity for both MOR and KOR, such as naltrexone, naloxone and nalmefene, could have a biphasic effect on striatal dopaminergic signalling (Rabiner et al. 2011) and it is possible that a MOR selective antagonist would modulate MID task anticipation BOLD responses in our data.

### Subjective effects of fentanyl

We previously showed ‘feel high’ and ‘drug effect’ VAS scores peaked 5–10 min following IV fentanyl administration (Zelaya et al. 2012). We, however, did not find any significant associations between these subjective effects of fentanyl 10 min post-administration and reward or loss anticipation BOLD contrast. We also found no correlations between CBF changes and subjective effects of fentanyl (Zelaya et al. 2012). Whilst our results may suggest that subjective intoxication is not associated with the fentanyl induced changes in BOLD we observed, it is also possible that there is significant variation in how subjective responses are rated by subjects reducing the replicability of the measure across participants. Unfortunately, there is a lack of human literature examining the neural correlates of the subjective effects of opioid intoxication which would allow us to better understand our findings.

### End Tidal CO_2_

There is evidence that changes in EtCO_2_, reflecting a change in blood CO_2_ concentration, modulate baseline BOLD signal (Cohen et al. 2002; Driver et al. 2017; Wise et al. 2007) and there is mixed evidence that increased EtCO_2_ may or may not be associated with changes in task based BOLD contrast (Corfield et al. 2001; Gauthier et al. 2011).

We found fentanyl reduced participants’ respiratory rate and increased EtCO_2_, reflecting the expected opioid-induced respiratory depression (Hill et al. 2020). We re-ran a priori analyses including ∆EtCO_2_ as a covariate. In ROI analyses we did not observe an effect of EtCO_2_ on BOLD contrast, suggesting that fentanyl induced changes in EtCO_2_ are not confounding our BOLD contrast comparisons in these regions.

Although there was evidence that the addition of ∆EtCO_2_ reduced the magnitude of these our significant results, importantly, we continued to observe significant effects of fentanyl on reward anticipation and loss anticipation independent of any effect of changes in EtCO_2_. Due to our small sample size we did not examine the effect of EtCO_2_ on BOLD signal, and this was not an aim of our study.

Our previous work using an arterial spin labelling (ASL) paradigm in the same subjects did not find a significant effect of changes in EtCO_2_ on cerebral blood perfusion in a number of ROIs (Zelaya et al. 2012), including the lingual gyrus and hippocampus where we observed a significant effect of fentanyl on anticipation BOLD contrast. Unlike our study, which showed regional effects of EtCo2 on BOLD response, other studies have shown more widespread effects of increased EtCO_2_ on BOLD signal (Corfield et al. 2001; Driver et al. 2017), which contrasts with our region specific results for the effect of fentanyl on BOLD signal. Furthermore, studies examining the effect EtCO_2_ on BOLD signal typically used substantially larger changes in EtCO_2_, for example 4 to 15 mmHg (Driver et al. 2017; Gauthier et al. 2011; Wise et al. 2007), compared with our mean changes of 1.6 mmHg following fentanyl administration (Table S8). Overall, our results indicate there is an effect of fentanyl on the MID anticipation BOLD responses, independent of fentanyl induced changes in EtCO_2_.

### Loss of scans and power

Unfortunately, a number of scan sessions were discarded from analyses for a range of reasons including excess motion and failure to respond appropriately to cues. This led to a reduction in power for some of our contrasts and also led some degree of unbalancing when comparing across contrasts as there were slightly different numbers of subjects (see Table S1). Power calculations to estimate required sample sizes were not carried out as part of the study design, but the sample sizes was based upon similar contemporary studies using the MID task (Cooper and Knutson 2008; Knutson et al. 2008). However, given the lack of adequate power calculations it is possible that our analyses are underpowered, which may result in an increase in the odds of both false negative and positive results (Button et al. 2013).

### Scan order effects

Whilst our scan order was randomised, there remains the possibility that after receiving their first IV fentanyl infusion, there could be an expectancy effect on subsequent scan sessions. There is evidence that even in experienced opioid users expectancy of receipt of IV heroin does not result in changes in dopamine release (Watson et al. 2014). Our participants were healthy volunteers with no use or abuse history of opioids and free of opioids on each session, confirmed with a urine drug screen. Despite this there may be differences between the first and second exposure to fentanyl. While there were differences between the second fentanyl session and the placebo scans in the anterior cingulate there was no evidence of a difference between the two fentanyl sessions. However, these sessions were embedded in pseudo-randomised order design and as discussed we may not have sufficient participants to be powered to investigate potential order effects.

### Male participants

Our data were collected from male participants only. There is evidence of sex differences in MID task fMRI neural responses (Dhingra et al. 2021) as well as regional brain MOR density (Kantonen et al. 2020). Therefore, further research would be required to verify if our current findings in males are generalisable to females.

### Changes in cerebral blood flow

As discussed above, we demonstrated increased CBF following IV fentanyl with ASL (Zelaya et al. 2012). CBF and the BOLD signal are closely linked (Simon and Buxton 2015) and it is possible that our significant results showing fentanyl induced increased in *reward > neutral* and *loss > neutral* anticipation BOLD contrast could be affected by background CBF changes following fentanyl administration. Increased CBF is more likely to be associated with a decrease in BOLD contrast (Simon and Buxton 2015) rather than the increased BOLD contrast we observed. Furthermore, not all regions with fentanyl induced CBF increases also had increased BOLD contrasts, for example the thalamus, which is part of the network of regions activated by the MID task (Knutson et al. 2001). However, due to the limited brain coverage of our ASL data we did not explore this further in our participant group.

## Conclusion

We did not observe any impact of MOR agonism with fentanyl on ‘wanting’ or ‘liking’ responses during the MID task in the striatum or within the broader mesocorticolimbic dopaminergic reward pathway. There is a wealth of evidence that opioidergic signalling plays a central role in pleasure and reward (Berridge and Kringelbach 2015; Le Merrer et al. 2009) therefore our results may support the hypothesis that the MID task is a method for probing the mechanisms of salience (Howes et al. 2020) rather than a ‘reward’ or ‘reward processing’ task as it is often referred to in the published literature.

Our data suggests that the MID task financial reward is insufficient to activate endogenous opioid signalling, indicated by the lack of effect of naloxone in our results. Using appropriate naturalistic rewards (e.g. feeding, sex, social contact) rather than a monetary reward may be more appropriate for probing reward-related opioidergic mechanisms in the human brain and help better understand characterise brain processes involved in the development of opioid dependence.

## Electronic supplementary material

Below is the link to the electronic supplementary material.

Supplementary Material 1

Supplementary Material 2

Supplementary Material 3

## Data Availability

Individual participant ROI data (beta weight values) for a priori reward and loss anticipation contrasts is included in the supplementary material section. Whole brain BOLD T-maps with significant results from our a priori reward and loss anticipation contrasts can be found on Neurovault (https://neurovault.org/collections/WRBNRUCP).

## References

[CR1] Aminoff EM, Kveraga K, Bar M (2013) The role of the parahippocampal cortex in cognition. Trends Cogn Sci 17(8):379–390. 10.1016/j.tics.2013.06.009 23850264 10.1016/j.tics.2013.06.009 PMC3786097

[CR2] Berridge KC, Kringelbach ML (2015) Pleasure systems in the brain. Neuron 86(3):646–664. 10.1016/j.neuron.2015.02.018 25950633 10.1016/j.neuron.2015.02.018 PMC4425246

[CR3] Bromberg-Martin ES, Matsumoto M, Hikosaka O (2010) Dopamine in Motivational Control: rewarding, aversive, and alerting. Neuron 68(5):815–834. 10.1016/j.neuron.2010.11.022 21144997 10.1016/j.neuron.2010.11.022 PMC3032992

[CR111] Buchel, C., Miedl, S., & Sprenger, C. (2018). Hedonic processing in humans is mediated by an opioidergic mechanism in a mesocorticolimbic system. eLife, 7, e39648.10.7554/eLife.39648 10.7554/eLife.39648 PMC6239433 30444488

[CR4] Burt JB, Helmer M, Shinn M, Anticevic A, Murray JD (2020) Generative modeling of brain maps with spatial autocorrelation. NeuroImage 220:117038. 10.1016/j.neuroimage.2020.117038 32585343 10.1016/j.neuroimage.2020.117038

[CR5] Button KS, Ioannidis JPA, Mokrysz C, Nosek BA, Flint J, Robinson ESJ, Munafò MR (2013) Power failure: why small sample size undermines the reliability of neuroscience. Nat Rev Neurosci 14(5):365–376. 10.1038/nrn3475 23571845 10.1038/nrn3475

[CR6] Castro DC, Berridge KC (2017) Opioid and orexin hedonic hotspots in rat orbitofrontal cortex and insula. Proc Natl Acad Sci USA 114(43):E9125–E9134. 10.1073/pnas.1705753114 29073109 10.1073/pnas.1705753114 PMC5664503

[CR7] Cavanna AE, Trimble MR (2006) The precuneus: a review of its functional anatomy and behavioural correlates. Brain 129(3):564–583. 10.1093/brain/awl004 16399806 10.1093/brain/awl004

[CR8] Clark SD, Abi-Dargham A (2019) The role of Dynorphin and the Kappa Opioid receptor in the Symptomatology of Schizophrenia: a review of the evidence. Biol Psychiatry 86(7):502–511. 10.1016/j.biopsych.2019.05.012 31376930 10.1016/j.biopsych.2019.05.012

[CR9] Cohen ER, Ugurbil K, Kim S-G (2002) Effect of basal conditions on the Magnitude and Dynamics of the blood oxygenation level-dependent fMRI response. J Cereb Blood Flow Metabolism 22(9):1042–1053. 10.1097/00004647-200209000-00002 10.1097/00004647-200209000-00002 12218410

[CR10] Colasanti A, Searle GE, Long CJ, Hill SP, Reiley RR, Quelch D, Erritzoe D, Tziortzi AC, Reed LJ, Lingford-Hughes AR, Waldman AD, Schruers KRJ, Matthews PM, Gunn RN, Nutt DJ, Rabiner EA (2012) Endogenous opioid release in the human brain reward System Induced by Acute Amphetamine Administration. Biol Psychiatry 72(5):371–377. 10.1016/j.biopsych.2012.01.027 22386378 10.1016/j.biopsych.2012.01.027

[CR11] Cooper JC, Knutson B (2008) Valence and salience contribute to nucleus accumbens activation. NeuroImage 39(1):538–547. 10.1016/j.neuroimage.2007.08.009 17904386 10.1016/j.neuroimage.2007.08.009 PMC2169259

[CR12] Corfield DR, Murphy K, Josephs O, Adams L, Turner R (2001) Does Hypercapnia-Induced Cerebral Vasodilation modulate the hemodynamic response to neural activation? NeuroImage 13(6):1207–1211. 10.1006/NIMG.2001.0760 11352626 10.1006/nimg.2001.0760

[CR13] Dahan A, Yassen A, Bijl H, Romberg R, Sarton E, Teppema L, Olofsen E, Danhof M (2005) Comparison of the respiratory effects of intravenous buprenorphine and fentanyl in humans and rats. Br J Anaesth 94(6):825–834. 10.1093/bja/aei145 15833777 10.1093/bja/aei145

[CR14] Demidenko MI, Weigard AS, Ganesan K, Jang H, Jahn A, Huntley ED, Keating DP (2021) Interactions between methodological and interindividual variability: how Monetary incentive Delay (MID) task contrast maps vary and impact associations with behavior. Brain Behav 11(5). 10.1002/brb3.2093 10.1002/brb3.2093 PMC8119872 33750042

[CR15] Dhingra I, Zhang S, Zhornitsky S, Wang W, Le TM, Li C-SR (2021) Sex differences in neural responses to reward and the influences of individual reward and punishment sensitivity. BMC Neurosci 22(1):12. 10.1186/s12868-021-00618-3 33639845 10.1186/s12868-021-00618-3 PMC7913329

[CR16] Driver ID, Wise RG, Murphy K (2017) Graded hypercapnia-calibrated BOLD: beyond the Iso-metabolic Hypercapnic Assumption. Front NeuroSci 11:276. 10.3389/fnins.2017.00276 28572755 10.3389/fnins.2017.00276 PMC5435758

[CR18] Eikemo M, Løseth GE, Johnstone T, Gjerstad J, Willoch F, Leknes S (2016) Sweet taste pleasantness is modulated by morphine and naltrexone. Psychopharmacology 233(21–22):3711–3723. 10.1007/s00213-016-4403-x 27538675 10.1007/s00213-016-4403-x

[CR17] Eikemo M, Biele G, Willoch F, Thomsen L, Leknes S (2017) Opioid modulation of Value-based decision-making in healthy humans. Neuropsychopharmacology 42(9):1833–1840. 10.1038/npp.2017.58 28294136 10.1038/npp.2017.58 PMC5520785

[CR19] Gauthier CJ, Madjar C, Tancredi FB, Stefanovic B, Hoge RD (2011) Elimination of visually evoked BOLD responses during carbogen inhalation: implications for calibrated MRI. NeuroImage 54(2):1001–1011. 10.1016/J.NEUROIMAGE.2010.09.059 20887792 10.1016/j.neuroimage.2010.09.059

[CR20] Gowin JL, Sloan ME, Kirk-Provencher KT, Rosenblatt SL, Penner AE, Stangl BL, Byrd ND, Swan JE, Ramchandani VA (2023) Opioid receptor antagonism and neural response to monetary rewards: pilot studies in light and heavy alcohol users. J Psychopharmacol 37(9):937–941. 10.1177/02698811231191707 37530456 10.1177/02698811231191707 PMC12951625

[CR21] Grill-Spector K, Kourtzi Z, Kanwisher N (2001) The lateral occipital complex and its role in object recognition. Vision Res 41(10–11):1409–1422. 10.1016/S0042-6989(01)00073-6 11322983 10.1016/s0042-6989(01)00073-6

[CR22] Grimm O, Nägele M, Küpper-Tetzel L, De Greck M, Plichta M, Reif A (2021) No effect of a dopaminergic modulation fMRI task by amisulpride and L-DOPA on reward anticipation in healthy volunteers. Psychopharmacology 238(5):1333–1342. 10.1007/s00213-020-05693-8 33140215 10.1007/s00213-020-05693-8 PMC8062334

[CR23] Hawkins PCT, Zelaya FO, O’Daly O, Holiga S, Dukart J, Umbricht D, Mehta MA (2021) The effect of risperidone on reward-related brain activity is robust to drug‐induced vascular changes. Hum Brain Mapp 42(9):2766–2777. 10.1002/hbm.25400 33666305 10.1002/hbm.25400 PMC8127149

[CR24] Hill R, Santhakumar R, Dewey W, Kelly E, Henderson G (2020) Fentanyl depression of respiration: comparison with heroin and morphine. Br J Pharmacol 177(2):254–265. 10.1111/bph.14860 31499594 10.1111/bph.14860 PMC6989952

[CR25] Howes OD, Hird EJ, Adams RA, Corlett PR, McGuire P (2020) Aberrant salience, Information Processing, and Dopaminergic Signaling in people at clinical high risk for psychosis. Biol Psychiatry 88(4):304–314. 10.1016/j.biopsych.2020.03.012 32430200 10.1016/j.biopsych.2020.03.012

[CR26] Kantonen T, Karjalainen T, Isojärvi J, Nuutila P, Tuisku J, Rinne J, Hietala J, Kaasinen V, Kalliokoski K, Scheinin H, Hirvonen J, Vehtari A, Nummenmaa L (2020) Interindividual variability and lateralization of µ-opioid receptors in the human brain. NeuroImage 217:116922. 10.1016/J.NEUROIMAGE.2020.116922 32407992 10.1016/j.neuroimage.2020.116922

[CR27] Karjalainen T, Seppälä K, Glerean E, Karlsson HK, Lahnakoski JM, Nuutila P, Jääskeläinen IP, Hari R, Sams M, Nummenmaa L (2019) Opioidergic regulation of emotional Arousal: a combined PET–fMRI study. Cereb Cortex 29(9):4006–4016. 10.1093/cercor/bhy281 30475982 10.1093/cercor/bhy281

[CR112] Knutson, B., Adams, C. M., Fong, G. W., & Hommer, D. (2001). Anticipation of increasing monetary reward selectively recruits nucleus accumbens. The Journal of Neuroscience : The Official Journal of the Society for Neuroscience, 21(16), RC159. 10.1523/JNEUROSCI.21-16-j0002.2001 PMC6763187 11459880

[CR29] Knutson B, Bjork JM, Fong GW, Hommer D, Mattay VS, Weinberger DR (2004) Amphetamine modulates human incentive Processing. Neuron 43(2):261–269. 10.1016/J.NEURON.2004.06.030 15260961 10.1016/j.neuron.2004.06.030

[CR28] Knutson B, Bhanji JP, Cooney RE, Atlas LY, Gotlib IH (2008) Neural responses to Monetary incentives in Major Depression. Biol Psychiatry 63(7):686–692. 10.1016/j.biopsych.2007.07.023 17916330 10.1016/j.biopsych.2007.07.023 PMC2290738

[CR30] Koechlin E (2011) Frontal Pole function: what is specifically human? Trends Cogn Sci 15(6):241. 10.1016/j.tics.2011.04.005 21601507 10.1016/j.tics.2011.04.005

[CR31] Koob GF, Volkow ND (2016) Neurobiology of addiction: a neurocircuitry analysis. Lancet Psychiatry 3(8):760–773. 10.1016/S2215-0366(16)00104-8 27475769 10.1016/S2215-0366(16)00104-8 PMC6135092

[CR32] Le Merrer J, Becker JAJ, Befort K, Kieffer BL (2009) Reward Processing by the Opioid System in the brain. Physiol Rev 89(4):1379–1412. 10.1152/physrev.00005.2009 19789384 10.1152/physrev.00005.2009 PMC4482114

[CR33] Leech R, Sharp DJ (2014) The role of the posterior cingulate cortex in cognition and disease. Brain 137(1):12–32. 10.1093/brain/awt162 23869106 10.1093/brain/awt162 PMC3891440

[CR34] Lemon RN (2008) Descending pathways in Motor Control. Annu Rev Neurosci 31(1):195–218. 10.1146/annurev.neuro.31.060407.125547 18558853 10.1146/annurev.neuro.31.060407.125547

[CR35] MacDonald AF, Billington CJ, Levine AS (2004) Alterations in food intake by opioid and dopamine signaling pathways between the ventral tegmental area and the shell of the nucleus accumbens. Brain Res 1018(1):78–85. 10.1016/j.brainres.2004.05.043 15262208 10.1016/j.brainres.2004.05.043

[CR36] Malén T, Karjalainen T, Isojärvi J, Vehtari A, Bürkner P-C, Putkinen V, Kaasinen V, Hietala J, Nuutila P, Rinne J, Nummenmaa L (2022) Atlas of type 2 dopamine receptors in the human brain: age and sex dependent variability in a large PET cohort. NeuroImage 255:119149. 10.1016/J.NEUROIMAGE.2022.119149 35367652 10.1016/j.neuroimage.2022.119149

[CR37] Massaccesi C, Korb S, Götzendorfer S, Chiappini E, Willeit M, Lundström JN, Windischberger C, Eisenegger C, Silani G (2024) Effects of dopamine and opioid receptor antagonism on the neural processing of social and nonsocial rewards. Hum Brain Mapp 45(4):e26645. 10.1002/hbm.26645 38445523 10.1002/hbm.26645 PMC10915723

[CR38] McGovern DJ, Polter AM, Prévost ED, Ly A, McNulty CJ, Rubinstein B, Root DH (2023) Ventral tegmental area glutamate neurons establish a mu-opioid receptor gated circuit to mesolimbic dopamine neurons and regulate opioid-seeking behavior. Neuropsychopharmacology 48(13):1889–1900. 10.1038/s41386-023-01637-w 37407648 10.1038/s41386-023-01637-w PMC10584944

[CR39] Milella MS, D’Ottavio G, De Pirro S, Barra M, Caprioli D, Badiani A (2023) Heroin and its metabolites: relevance to heroin use disorder. Translational Psychiatry 13(1):120. 10.1038/s41398-023-02406-5 37031205 10.1038/s41398-023-02406-5 PMC10082801

[CR40] Mitchell JM, O’Neil JP, Janabi M, Marks SM, Jagust WJ, Fields HL (2012) Alcohol consumption induces endogenous opioid release in the human orbitofrontal cortex and nucleus accumbens. Sci Transl Med 4(116):116ra6. 10.1126/scitranslmed.3002902 22238334 10.1126/scitranslmed.3002902

[CR41] Murphy A, Nestor LJ, McGonigle J, Paterson L, Boyapati V, Ersche KD, Flechais R, Kuchibatla S, Metastasio A, Orban C, Passetti F, Reed L, Smith D, Suckling J, Taylor E, Robbins TW, Lingford-Hughes A, Nutt DJ, Deakin JF, Elliott R (2017) Acute D3 antagonist GSK598809 selectively enhances neural response during Monetary reward anticipation in drug and alcohol dependence. Neuropsychopharmacology 42(5):1049–1057. 10.1038/npp.2016.289 28042871 10.1038/npp.2016.289 PMC5423526

[CR42] Nestor LJ, Murphy A, McGonigle J, Orban C, Reed L, Taylor E, Flechais R, Paterson LM, Smith D, Bullmore ET, Ersche KD, Suckling J, Tait R, Elliott R, Deakin B, Rabiner I, Lingford-Hughes A, Nutt DJ, Sahakian B, Robbins TW (2017) Acute naltrexone does not remediate fronto-striatal disturbances in alcoholic and alcoholic polysubstance-dependent populations during a monetary incentive delay task. Addict Biol 22(6):1576–1589. 10.1111/adb.12444 27600363 10.1111/adb.12444

[CR43] Nutt DJ, Lingford-Hughes A, Erritzoe D, Stokes PRA (2015) The dopamine theory of addiction: 40 years of highs and lows. Nat Rev Neurosci 16(5):305–312. 10.1038/nrn3939 25873042 10.1038/nrn3939

[CR44] Oldham S, Murawski C, Fornito A, Youssef G, Yücel M, Lorenzetti V (2018) The anticipation and outcome phases of reward and loss processing: a neuroimaging meta-analysis of the monetary incentive delay task. Hum Brain Mapp 39(8):3398–3418. 10.1002/hbm.24184 29696725 10.1002/hbm.24184 PMC6055646

[CR45] Palejwala AH, Dadario NB, Young IM, O’Connor K, Briggs RG, Conner AK, O’Donoghue DL, Sughrue ME (2021) Anatomy and White Matter connections of the Lingual Gyrus and Cuneus. World Neurosurg 151:e426–e437. 10.1016/j.wneu.2021.04.050 33894399 10.1016/j.wneu.2021.04.050

[CR46] Pathan H, Williams J (2012) Basic opioid pharmacology: an update. Br J Pain 6(1):11–11 26516461 10.1177/2049463712438493 PMC4590096

[CR47] Pattinson KTS, Wise RG (2016) Imaging the Respiratory Effects of Opioids in the Human Brain. In R. C. Roach, P. H. Hackett, & P. D. Wagner (Eds.), Hypoxia: Translation in Progress (pp. 145–156). Springer US. 10.1007/978-1-4899-7678-9_10 10.1007/978-1-4899-7678-9_10 27343094

[CR48] Pizzagalli DA, Smoski M, Ang Y-S, Whitton AE, Sanacora G, Mathew SJ, Nurnberger J, Lisanby SH, Iosifescu DV, Murrough JW, Yang H, Weiner RD, Calabrese JR, Goodman W, Potter WZ, Krystal AD (2020) Selective kappa-opioid antagonism ameliorates anhedonic behavior: evidence from the fast-fail trial in Mood and anxiety Spectrum disorders (FAST-MAS). Neuropsychopharmacology 45(10):1656–1663. 10.1038/s41386-020-0738-4 32544925 10.1038/s41386-020-0738-4 PMC7419512

[CR49] Quelch DR, Mick I, McGonigle J, Ramos AC, Flechais RSA, Bolstridge M, Rabiner E, Wall MB, Newbould RD, Steiniger-Brach B, van den Berg F, Boyce M, Østergaard Nilausen D, Breuning Sluth L, Meulien D, von der Goltz C, Nutt D, Lingford-Hughes A (2017) Nalmefene reduces reward anticipation in Alcohol Dependence: an experimental functional magnetic resonance imaging study. Biol Psychiatry 81(11):941–948. 10.1016/j.biopsych.2016.12.029 28216062 10.1016/j.biopsych.2016.12.029

[CR50] Rabiner EA, Beaver J, Makwana A, Searle G, Long C, Nathan PJ, Newbould RD, Howard J, Miller SR, Bush MA, Hill S, Reiley R, Passchier J, Gunn RN, Matthews PM, Bullmore ET (2011) Pharmacological differentiation of opioid receptor antagonists by molecular and functional imaging of target occupancy and food reward-related brain activation in humans. Mol Psychiatry 16(8):826–835. 10.1038/mp.2011.29 21502953 10.1038/mp.2011.29 PMC3142667

[CR51] Reeves KC, Shah N, Muñoz B, Atwood BK (2022) Opioid receptor-mediated regulation of neurotransmission in the brain. Front Mol Neurosci 15:919773. 10.3389/fnmol.2022.919773 35782382 10.3389/fnmol.2022.919773 PMC9242007

[CR52] Schott BH, Minuzzi L, Krebs RM, Elmenhorst D, Lang M, Winz OH, Seidenbecher CI, Coenen HH, Heinze H-J, Zilles K, Düzel E, Bauer A (2008) Mesolimbic functional magnetic resonance imaging activations during reward anticipation correlate with reward-related ventral striatal dopamine release. J Neuroscience: Official J Soc Neurosci 28(52):14311–14319. 10.1523/JNEUROSCI.2058-08.2008 10.1523/JNEUROSCI.2058-08.2008 PMC6671462 19109512

[CR53] Sescousse G, Barbalat G, Domenech P, Dreher J-C (2013) Imbalance in the sensitivity to different types of rewards in pathological gambling. Brain 136(8):2527–2538. 10.1093/brain/awt126 23757765 10.1093/brain/awt126

[CR54] Shatalina E, Ashok AH, Wall MB, Nour MM, Myers J, Reis Marques T, Rabiner EA, Howes OD (2023) Reward processing in schizophrenia and its relation to Mu opioid receptor availability and negative symptoms: a [11 C]-carfentanil PET and fMRI study. NeuroImage: Clin 39:103481. 10.1016/j.nicl.2023.103481 37517175 10.1016/j.nicl.2023.103481 PMC10400918

[CR55] Simon AB, Buxton RB (2015) Understanding the dynamic relationship between cerebral blood flow and the BOLD signal: implications for quantitative functional MRI. NeuroImage 116:158–167. 10.1016/j.neuroimage.2015.03.080 25862267 10.1016/j.neuroimage.2015.03.080 PMC4468003

[CR56] Stanley TH (1992) The history and development of the fentanyl series. J Pain Symptom Manag 7(3):S3–S7. 10.1016/0885-3924(92)90047-L 10.1016/0885-3924(92)90047-l 1517629

[CR57] Stanley TH, Webster LR (1978) Anesthetic requirements and Cardiovascular effects of FentanyI-Oxygen and FentanyI-Diazepam-oxygen anesthesia in Man. Anesth Analg 57:411–416 568401 10.1213/00000539-197807000-00008

[CR58] Stevens FL, Hurley RA, Taber KH (2011) Anterior cingulate cortex: unique role in cognition and emotion. J Neuropsychiatry Clin Neurosci 23(2). 10.1176/jnp.23.2.jnp121 10.1176/jnp.23.2.jnp121 21677237

[CR59] Sun L, Lukkarinen L, Putkinen V, Karlsson HK, Hirvonen J, Tiihonen J, Lauerma H, Scott S, Nummenmaa L (2022) Mu-opioid receptor system modulates responses to vocal bonding and distress signals in humans. Philosophical Trans Royal Soc B: Biol Sci 377(1863):20210181. 10.1098/rstb.2021.0181 10.1098/rstb.2021.0181 PMC9489292 36126675

[CR60] Turton S, Paterson LM, Myers JF, Mick I, Lan C-C, McGonigle J, Bowden-Jones H, Clark L, Nutt DJ, Lingford-Hughes AR (2024) Exploratory study of associations between monetary reward anticipation brain responses and mu-opioid signalling in alcohol dependence, gambling disorder and healthy controls. NeuroImage: Rep 4(3):100211. 10.1016/j.ynirp.2024.100211 39345862 10.1016/j.ynirp.2024.100211 PMC11427764

[CR61] Tuulari JJ, Tuominen L, de Boer FE, Hirvonen J, Helin S, Nuutila P, Nummenmaa L (2017) Feeding releases endogenous opioids in humans. J Neuroscience: Official J Soc Neurosci 37(34):8284–8291. 10.1523/JNEUROSCI.0976-17.2017 10.1523/JNEUROSCI.0976-17.2017 PMC6596790 28747384

[CR62] Urban NBL, Slifstein M, Meda S, Xu X, Ayoub R, Medina O, Pearlson GD, Krystal JH, Abi-Dargham A (2012) Imaging human reward processing with positron emission tomography and functional magnetic resonance imaging. Psychopharmacology 221(1):67–77. 10.1007/s00213-011-2543-6 22052081 10.1007/s00213-011-2543-6

[CR63] Van Steenbergen H, Eikemo M, Leknes S (2019) The role of the opioid system in decision making and cognitive control: a review. Cogn Affect Behav Neurosci 19(3):435–458. 10.3758/s13415-019-00710-6 30963411 10.3758/s13415-019-00710-6 PMC6599188

[CR64] Volkow ND, Michaelides M, Baler R (2019) The neuroscience of drug reward and addiction. Physiol Rev 99(4):2115–2140. 10.1152/physrev.00014.2018 31507244 10.1152/physrev.00014.2018 PMC6890985

[CR65] Watson BJ, Taylor LG, Reid AG, Wilson SJ, Stokes PR, Brooks DJ, Myers JF, Turkheimer FE, Nutt DJ, Lingford-Hughes AR (2014) Investigating expectation and reward in human opioid addiction with [11 C]raclopride PET. Addict Biol 19(6):1032–1040. 10.1111/adb.12073 23829344 10.1111/adb.12073 PMC4282066

[CR66] Will MJ, Pratt WE, Kelley AE (2006) Pharmacological characterization of high-fat feeding induced by opioid stimulation of the ventral striatum. Physiol Behav 89(2):226–234. 10.1016/j.physbeh.2006.06.008 16854442 10.1016/j.physbeh.2006.06.008

[CR67] Wilson RP, Colizzi M, Bossong MG, Allen P, Kempton M, Bhattacharyya S, Bhattacharyya S (2018) The neural substrate of reward anticipation in Health: a Meta-analysis of fMRI findings in the Monetary incentive Delay Task. Neuropsychol Rev 28(4):496–506. 10.1007/s11065-018-9385-5 30255220 10.1007/s11065-018-9385-5 PMC6327084

[CR68] Wise RG, Pattinson KTS, Bulte DP, Chiarelli PA, Mayhew SD, Balanos GM, O’Connor DF, Pragnell TR, Robbins PA, Tracey I, Jezzard P (2007) Dynamic forcing of end-tidal carbon dioxide and oxygen applied to functional magnetic resonance imaging. J Cereb Blood Flow Metab 27(8):1521–1532. 10.1038/sj.jcbfm.9600465 17406659 10.1038/sj.jcbfm.9600465

[CR69] Zelaya FO, Zois E, Muller-Pollard C, Lythgoe DJ, Lee S, Andrews C, Smart T, Conrod P, Vennart W, Williams SCR, Mehta MA, Reed LJ (2012) The response to rapid infusion of fentanyl in the human brain measured using pulsed arterial spin labelling. MAGMA 25(2):163–175. 10.1007/s10334-011-0293-4 22113518 10.1007/s10334-011-0293-4

[CR70] Zeng J, Yan J, Cao H, Su Y, Song Y, Luo Y, Yang X (2022) Neural substrates of reward anticipation and outcome in schizophrenia: a meta-analysis of fMRI findings in the monetary incentive delay task. Translational Psychiatry 12(1):448. 10.1038/s41398-022-02201-8 36244990 10.1038/s41398-022-02201-8 PMC9573872

[CR71] Zhang J, Song C, Dai J, Li L, Yang X, Chen Z (2022) Mechanism of opioid addiction and its intervention therapy: focusing on the reward circuitry and mu-opioid receptor. MedComm 3(3):e148. 10.1002/mco2.148 35774845 10.1002/mco2.148 PMC9218544

