# Supplementary material for: Opioidergic modulation of monetary incentive delay fMRI responses
Source: Psychopharmacology (Berl). 2025 Feb 12;242(8):1743–56. doi: 10.1007/s00213-025-06753-7 (PMC12296811; doi:10.1007/s00213-025-06753-7)
Supplement: Supplementary file 3 — Supplementary Material 3 [file 213_2025_6753_MOESM3_ESM.docx]

**OPIOIDERGIC MODULATION OF MONETARY INCENTIVE DELAY FMRI RESPONSES**

**PSYCHOPHARMACOLOGY**

Dr Samuel Turton^1,2^, Dr Peter CT Hawkins^1^, Dr Christopher Muller-Pollard^3^, Prof Evangelos Zois^4^, Prof Patricia Conrod^5^, Dr Fernando Zelaya^1^ and Prof Mitul A Mehta^1^

1. Institute of Psychiatry, Psychology and Neuroscience, King's College London, London, UK.
2. Division of Psychiatry, Imperial College London, London, UK
3. Neurodevelopmental Service Sussex Partnership NHS Foundation Trust, UK
4. IU International University of Applied Sciences, Bad Honnef, Germany
5. Department of Psychiatry, Université de Montreal, CHU Ste Justine Hospital, Montreal, QC, Canada.

Communicating Author – [samuel.turton@kcl.ac.uk](mailto:samuel.turton@kcl.ac.uk)

**SUPPLEMENTARY METHODS:**

Study Subjects

A total of 27 right-handed males were initially recruited into the study. They had no previous psychiatric or neurological diagnoses, and no significant current or past medical history. These were determined by clinical assessment and completing the Structural Clinical Interview of the Diagnostic Statistical Tool for Mental Disorders (First et al., 2002). All participants consumed <21 units of alcohol/week and <10 cigarettes/month.

Three participants were excluded from the analysis (1 participant completed only 1 scan, 1 participant was excluded due to problems with spatial co-registration of the BOLD fMRI data and 1 participant was excluded due to excessive motion during all scans sessions). Of the remaining 24 participants (mean age 25.5 years, range 19 to 36 years), some individual scans were either missing (i.e. due to acquisition errors) or excluded due to excessive motion (see *Pre-Processing* section for details). Furthermore, some scans were excluded from certain contrasts, for example due to non-responses to neutral cues or not enough misses for a win vs. lose feedback contrasts. The total number of scans available for each contrast is detailed in *Table S1*.

All participants provided written informed consent to participate, and the study was approved by Guy’s and St. Thomas’ Hospital Research Ethics Committee (06/Q0704/24).

Study and scanning procedures

Participants attended for a screening session and four scanning sessions at least 1 week apart. On each visit, participants underwent a brief physical screening including vital signs and completed urine drug screening (Triage® panel - www.biosite.com) alcohol breath testing (Alco-Sensor FST® hand held tester - [www.intox.com](http://www.intox.com)).

Participants were positioned within the General Electric Excite HDX 3.0 Tesla scanner (Milwaukee, Wisconsin, USA) MRI scanner and received either one of two doses of fentanyl (50 μg/70 kg), naloxone (400 μg) or placebo (0.9% saline) in a volume of 10 ml over 1 min administered via the antecubital vein. The administration was using a randomised, double-blinded Williams’ square design. Participants did not observe drug administration whilst in the scanner.

Participants completed a task training session prior to scanning. Approximately 15 mins after the IV infusion participants completed an MID task consisting of high- and low-reward and loss cues and a neutral cue. Continuous end tidal CO_2_ (EtCO_2_) and respiratory rate date were collected during scanning. Participants completed subjective ratings of drug effects using a visual analogue scale (VAS) during the scan (see *Subjective effects of study medication* section for details).

Image acquisition

All scans were conducted on a General Electric Excite HDX 3.0 Tesla scanner (Milwaukee, Wisconsin, USA). The following sequences were collected on each of the four scan visits.

MID task functional scans were carried out using a temporal series of Gradient-recalled- echo planar imaging (GE-EPI) whole brain scans, comprising of 27 slices with a thickness of 5 mm (TR = 2000 ms, TE = 30, flip angle = 80 degrees, number of volumes = 720, FoV = 240 mm, slice gap 5.5mm).

T1-weighted 3D fast spoiled gradient echo (FSPGR) was collected for use in spatial normalisation of the functional data, comprising of 196 slices with a thickness of 1.1 mm (TR = 6.62 ms, TE = 2.81 ms, flip angle = 20 degrees, FoV = 280 mm, slice gap 1.1mm). T1 and T2-weighted scans were reviewed by a neuroradiologist to exclude the presence of any brain tissue lesions. The single T1-weighted scan with the least artefact was selected to be used in spatial normalisation for all subsequent analysis for each participant.

High resolution gradient echo (HRGE) for use in spatial normalisation of the functional data, comprising of 43 slices with a thickness of 3 mm (TR = 3000 ms, TE = 30 ms, flip angle = 90 degrees, FoV = 240 mm, slice gap 3.3 mm).

Pre-processing

Pre-processing was carried out in SPM12, running in Matlab 9.5.0.1586782. The origin of functional and structural images was reset to the anterior commissure-posterior commissure line. Functional images were slice-time corrected (reference slice: 14) and six rigid body motion parameters (translation and rotation in x, y, z) were derived along with framewise displacement. Each subject’s functional volumes were then realigned to a mean image of all four sessions.

The HRGE images were linearly co-registered to the mean functional image from the realignment step and brain extracted. The brain extracted T1 image was then linearly co-registered to the HRGE brain extracted image, using the normalised mutual information objective function in SPM, and a DARTEL (Diffeomorphic anatomical registration through exponentiated lie algebra (Ashburner, 2007)) template was created from the T1-weighted images. The realigned and co-registered functional volumes were resliced to original voxel sizes and the DARTEL flow fields applied to warp the data into MNI space. Normalised images were smoothed using an 8mm FWHM kernel.

The addition HRGE co-registration step was required due to issues with significant mis-alignment when directly co-registering the T1 image to the functional image. All co-registered images were visually inspected for quality control.

Motion and framewise displacement parameters estimated during the realignment process were added as regressors in the first-level design matrix (Siegel et al., 2014). Any volumes with displacement of 1mm or more were flagged and marked with a 3-TR regressor (to include the volumes either side) in the first level design matrix (Power et al., 2012). The realignment parameters were visually inspected and any time-series for which the maximum detected translation from the first volume was greater than the dimensions of one voxel, or those which indicated stimulus correlated movement, were flagged for exclusion.

Any scan that required more than 10% of the volumes of the full run being regressed out, or a single volume to volume movement of >3mm or had a total movement of >100 mm resulted in that scan participant being removed from the analysis. One participant was completely excluded from further analysis on these criteria due to excess motion in all 4 scanning sessions and 3 individual scans from separate participants were also excluded (1 fentanyl, 1 naloxone and 1 placebo).

MID Task and first-level modelling

Our MID task version lasted 24 minutes and consisted of high- and low-reward cues (£2.00 and £0.20 respectively), high- and low-loss cues (-£2.00 and -£0.20) and a neutral cue, all requiring an active response from the participant. Each cue was presented 24 times,

except the neutral cue which was presented 48 times. Each trial lasted 10 seconds with a variable anticipation period, ranging from 4060 to 4500 ms, followed by the target also of varying duration of up to 300 ms which calibrated according to the participant’s performance during the task. There was followed by an outcome period where the participant was informed whether they ‘hit’ or ‘missed’ the target, any win or loss amount and their total winnings. Four separate ‘playlists’ with different order of the trials were randomised to minimise order or learning effects from repeating the task on four sessions.

The MID task was modelled as outlined by Abler et al. (2007). Five cue regressors (high-reward, low-reward, high-loss, low-loss and neutral) were defined for the anticipatory period depending on the cue presented. The target was defined by a single regressor of 500 ms.

Nine outcome regressors (high-reward win, low-reward win, high-reward lose, low-reward lose, high-loss win, low-loss win, high-loss lose, low-loss lose and neutral) were defined depending on the cue type and outcome and set for a fixed period (1450 ms). Motion and framewise displacement parameters estimated during the realignment process were also added resulting in the model consisting of the fourteen task conditions above and seven movement-related regressors.

Our two a priori anticipation period contrasts of interest were defined as combined-reward>neutral and combined-loss>neutral. Different levels of reward and loss trials were combined to increase the number of trials and improve the power of our a priori analysis. We also explored an additional 6 anticipation contrasts: high-reward>neutral, low-reward>neutral, high-reward>low-reward, high-loss>neutral, low-loss>neutral and high-loss>low-loss, to explore if there was a differential effect of naltrexone or fentanyl on different value reward and loss trials. We also explored 3 outcome contrasts: reward trial successful win>lose, loss trial successful win>lose and combined reward and loss trial successful win>lose.

MID task performance and behavioural data

Performance related criteria were set to ensure only data from participants who were actively and appropriately engaged in the task were included in the final analysis. If the participant pressed the button during the presentation of the fixation cross or within 100 ms of the presentation of the target (an unrealistic reaction time), the trial was defined as a regressor of no interest. When participants failed to make a response to an active trial (i.e. no button press was attempted), the trial also defined as a regressor of no interest.

An attempted response rate (i.e. a button press within the entire 500 ms response window, regardless of win or lose) >66% was also required for a participant’s MID task to be included in the final analysis. Any individual task runs with a response rate less than <33% in a particular scan were excluded from further analysis involving that drug condition in the contrast. This was only an issue with neutral trial responses and resulted in 10 individual scans across 5 participants (2 fentanyl-1, 2 fentanyl-2, 4 naloxone and 2 placebo) being excluded from contrasts including the neutral anticipation condition (i.e. combined-reward>neutral anticipation). This was not indicative of a broader issue with neutral trial responses as the majority of scans (>80%) had a response rate of at least 66% to neutral trials.

There were also a number of participant scans with a low number of total misses (less than 10 misses out of 48 trials) for reward or loss trials, and these were excluded from relevant outcome contrasts, for example combined-reward win>lose outcome.

Performance and behavioural data; accuracy, reaction time, total winnings and total interframe movement, were output from our pre-processing and analysed in R (version 4.2.1 , 23^rd^ Jun 2022) using anova (res.aov) and post-hoc t-testing if appropriate.

Contrasts of interest

Our a priori contrasts of interest were weighted to explore main effect of anticipation of reward: High-Reward & Low-Reward > Neutral Cue [0.5, 0.5, −1] and anticipation of loss: High-Loss & Low-Loss > Neutral Cue [0.5, 0.5, −1].

We explored the differential effects of fentanyl and naloxone and on reward and loss anticipation with the following contrasts: High-Reward > Neutral Cue [1, -1], Low-Reward > Neutral Cue [1, -1], High-Reward > Low-Reward [1, -1], High-Loss > Neutral Cue [1, -1], Low-Reward > Neutral Cue [1, -1], High-Loss > Low-Loss [1, -1].

We also explored the effects of fentanyl and naloxone on Reward and Loss trial outcomes: High-Reward win & Low-Reward win > High-Reward lose & Low-Reward lose [0.5, 0.5, -0.5, -0.5], High-Loss win & Low-Loss win > High-Loss lose & Low-Loss lose [0.5, 0.5, -0.5, -0.5] and High-Reward win & Low-Reward win & High-Loss win & Low-Loss win > High-Reward lose & Low-Reward lose & High-Loss lose & Low-Loss lose [0.25, 0.25, 0.25, 0.25, -0.25, -0.25, -0.25, -0.25].

Second-level analysis

Second-level analysis was carried out in SPM12 using a random effects model and including an F-test and post-hoc paired t-tests. The two fentanyl scan sessions; fentanyl-1 and fentanyl-2, were combined into a single condition for the second-level analysis. We did this as we were more interested in the effect of fentanyl compared with naloxone and placebo, than the differential effects of the first and second doses of fentanyl. Combining the scans increased the power of the *fentanyl > placebo* and *fentanyl > naloxone* contrasts by increasing the amount of data for each participant, and also addressed the issue with some participants being excluded from second-level fentanyl contrasts as they were missing a single fentanyl scan, for example due to excess movement, or non-response to neutral cues.

We did, however, also re-run all second-level analyses with fentanyl-1 and fentanyl-2 as separate conditions to examine differential effects between these two doses.

Subjective effects of study medication

Participants completed visual analogue scales (VAS) to assess subjective effects of the study medication at -10 mins prior to and +1, +8, +15, +45 and +55 mins following the IV infusion as previously reported by Zelaya et al., (2012). The scales were presented within the scanner and participants used an analogue joystick to select a number from the scale which was present on a screen for 5 s. The following items were presented ‘Do you feel a drug effect’, ‘Do you feel high’, ‘Do you feel drowsy’ and ‘Do you feel spaced out’. For the purposes of our analyses we focussed on ‘drug effect’ and ‘feel high’ scores completed prior to starting the MID task (+10 mins following infusion).

Differences in VAS scores between different scan conditions were analysed in R (version 4.2.1), 23^rd^ Jun 2022) using ANOVA (res.aov) and post-hoc t-testing as appropriate.

To assess associations between subjective effects of fentanyl and combined High-Reward & Low-Reward > Neutral anticipation and combined High-Loss & Low-Loss > Neutral anticipation contrasts, the VAS scores were included in the second-level models as covariates. This was examined in fentanyl-1 and fentanyl-2 conditions separately and then a combined-fentanyl condition.

ROI definition

Five bilateral a priori regions of interest (ROIs) were defined to explore the effects of fentanyl and naloxone on MID task BOLD responses: ventral striatum, caudate, putamen, anterior cingulate and anterior insula (Chen et al., 2022; Oldham et al., 2018; Wilson et al., 2018). ROIs were defined using the Harvard-Oxford (HO) Cortical and Subcortical probabilistic atlases and were thresholded at >50% likelihood to eliminate overlap between adjacent regions, which was particularly an issue in the striatum.

These regions were then grey matter masked. The anterior insular was defined as any volume of the HO cortical atlas insular cortex anterior to the Y=0 plane in Montreal Neurological Institute (MNI) space.

ROI mean beta estimates for each contrast (see above) were extracted from using the SPM12 MarsBar plugin. ROI beta estimates were analysed with a linear mixed effects model using the lme4 package in R (version 4.2.1 , 23^rd^ Jun 2022), for each contrast separately with Drug and ROI as fixed factors and Subject as a random factor. These analyses were primarily used to examine the within-subject effect of drug across all ROIs. Post‐hoc pairwise comparisons to examine differences within individual ROIs between Drug conditions were carried out using the paired t-test function within the ‘rstatix’ package.

EtCO2

Respiratory rate (RR) and end tidal CO_2_ (EtCO_2_) were recorded during the scan. Upon inspection of the data record, EtCO_2_ data were missing for 10 participants; 2 not-recorded, 7 missing 1 scan’s data, 1 missing 2 scan’s data. Data was missing due to technical failures of the capnograph, and for 2 participants there no recorded data available at the time of analysis. Mean EtCO_2_ values were calculated as a baseline 10mins prior to the study drug infusion and for the duration of the MID scan. These values where then used to calculate a proportional change in EtCO_2_ (∆EtCO_2_) during the MID scan compared to baseline.

RR data was missing for 6 participants due to data collection errors and 2 participants had no data available at the time of analysis.

To explore the effects of EtCO_2_ on our reward and loss anticipation BOLD contrasts, ∆EtCO_2_ was added as a covariate to the linear mixed effects model in R and the SPM second level model in our ROI and whole-brain analyses respectively.

Mu-opioid receptor PET maps

To examine the relative mu opioid receptor density in regions or volumes with significant MID task BOLD contrast, regional mean [^11^C]carfentanil BP_ND_ values were extracted from a publicly available PET atlas in 204 heathy individuals (Kantonen et al., 2020) which was downloaded from neurovault (<https://neurovault.org/collections/GCELSAIA>). The PET atlas consists of voxel-wise [^11^C]carfentanil BP_ND_ values. Mean BP_ND_ values were extracted for each region in the Harvard-Oxford cortical and subcortical atlases using the SPM12 MarsBar toolbox. Each region in the atlas was thresholded at a minimum 25% probability, and a combination of left and right (i.e. bilateral) regions were used.

Mean [^11^C]carfentanil BP_ND_ values for each significant cluster from reward and loss anticipation BOLD contrast whole brain analyses were also extracted using SPM12 MarsBar toolbox using a binarized mask consisting of the entire volume of each individual cluster.

Regional correlations between [^11^C]carfentanil BP_ND_ and fentanyl>placebo reward anticipation and loss anticipation were carried out using BrainSMASH, a method to address the issue of spatial autocorrelation in these data which can result in false positives (Burt et al., 2020). These analyses were performed with Python (3.8.16) in Jupyter notebooks, SciPy (version 1.10.1) (Virtanen et al., 2020) and BrainSMASH (version 0.11.0). Regions were defined using the 500 ROI Cammoun parcellation (Cammoun et al., 2012). Pearson’s correlation coefficient analyses, corrected for spatial autocorrelation, were carried out between regional mean [^11^C]carfentanil BP_ND_ values and mean T-values across subjects from the following whole brain contrasts:

1. Combined fentanyl > placebo, combined high- & low-reward>neutral
2. Combined fentanyl > placebo, combined high- & low-reward>neutral

**Table S1** – Participant numbers included in each a priori and exploratory contrast

| **Contrast** | **Fent1 vs Fent2** | **Fent1 vs Plac** | **Fent1 vs Nalox** | **Fent2 vs Plac** | **Fent2 vs Nalox** | **Comb-Fent vs Plac** | **Comb-Fent vs Nalox** | **Plac vs Nalox** |
| --- | --- | --- | --- | --- | --- | --- | --- | --- |
| **Anticipation** | | | | | | | | |
| Combined-reward>neutral | 20 | 20 | 17 | 21 | 17 | 21 | 17 | 17 |
| Combined-loss>neutral | 20 | 20 | 17 | 21 | 17 | 21 | 17 | 17 |
| High- reward>neutral | 20 | 20 | 17 | 21 | 17 | 21 | 17 | 17 |
| Low-reward>neutral | 20 | 20 | 17 | 21 | 17 | 21 | 17 | 17 |
| High-loss>neutral | 20 | 20 | 17 | 21 | 17 | 21 | 17 | 17 |
| Low-loss>neutral | 20 | 20 | 17 | 21 | 17 | 21 | 17 | 17 |
| High-Reward>low-Reward | 22 | 22 | 22 | 23 | 22 | 23 | 22 | 22 |
| High-Loss>low-loss | 22 | 22 | 22 | 23 | 22 | 23 | 22 | 22 |
| **Outcome** | | | | | | | | |
| Combined-reward win>lose | 17 | 17 | 15 | 20 | 15 | 20 | 15 | 15 |
| Combined-loss win>lose | 17 | 17 | 17 | 19 | 17 | 21 | 17 | 17 |
| Combined-reward and loss win>lose | 18 | 18 | 16 | 21 | 16 | 22 | 16 | 16 |

**SUPPLEMENTARY RESULTS:**

**Table S2** - Task behavioural data (mean ±SD)

|  | **Fentanyl-1** | **Fentanyl-2** | **Naloxone** | **Placebo** | **Repeated measure ANOVA p-value** |
| --- | --- | --- | --- | --- | --- |
| Task Accuracy  (hit %) | 64.8  (±7.7) | 64.1  (±9.2) | 62  (±6.54) | 61.8  (±7.29) | 0.264 |
| Task reaction time (ms) | 249  (±25) | 248  (±27) | 251  (±25) | 251  (±23) | 0.965 |
| Total Winnings (£) | 31.9  (±17.7) | 29.0 (±15.6) | 35.0  (±10.6) | 28.7 (±12.5) | 0.359 |

**Table S3** – Task related movement (mean ±SD)

|  | **Fentanyl-1** | **Fentanyl-2** | **Naloxone** | **Placebo** | **Repeated measure ANOVA p-value** |
| --- | --- | --- | --- | --- | --- |
| Total interframe movement (mm) | 54.1  (±18.4) | 51.5  (±14.0) | 58.7  (±15.4) | 52.6  (±20.4) | 0.290 |

**Table S4** – Mixed-model results ROI analyses showing exploratory anticipation and outcome contrasts for the within-subject effect of ‘drug’

| **Contrast** | **Df** | **F-value** | **p-value** |
| --- | --- | --- | --- |
| ***Anticipation*** | | | |
| High-reward>neutral | 3, 358.9 | 6.1 | **<0.001** |
| High-loss>neutral | 3, 358.3 | 3.4 | 0.018 |
| High-reward>low-reward | 3, 408.9 | 4.4 | **0.004** |
| High-loss>low-loss | 3, 406.3 | 2.4 | 0.066 |
| Low-reward>neutral | 3, 362.2 | 5.2 | **0.002** |
| Low-loss>neutral | 3, 360.1 | 9.9 | **<0.001** |
| ***Outcome*** | | | |
| Combined-reward trail win>lose | 3, 332.0 | 2.4 | 0.071 |
| Combined loss trial win>lose | 3, 339.7 | 4.6 | **0.004** |
| Combined reward and loss win>lose | 3, 306.1 | 2.4 | 0.065 |

*Bonferroni corrected p<0.005*

**Figure S1** – Main effects of task following placebo administration including MNI z-coordinate and presented in neurological orientation (i.e. image left is subject’s left):
**A)** Reward Anticipation

**

**B)** Combined-Loss > Neutral Anticipation

**

**Table S5** – a priori whole brain analysis results: combined-reward>neutral and combined-loss >neutral anticipation. Including cluster regional details from Harvard-Oxford Cortical and Subcortical Structural Atlases. Local cluster maxima locations in bold

| **Combined-Reward > Neutral anticipation: Fentanyl > Placebo** | | | | | | | |
| --- | --- | --- | --- | --- | --- | --- | --- |
| Cluster-level p-FWE | Cluster size  (vox) | Local maxima  t-stat | MNI coordinates (mm) | | | Cluster Regional Details | |
|  |  |  | X | Y | Z | Local Maxima | Other regions within cluster |
| <0.001  (Cluster 1) | 10990 | 5.45 | 8 | -48 | 0 | **Right lingual gyrus** | - Left ligual, posterior cingulate, precentral & middle frontal gyri & central opercular & precuneus cortex  - Bilateral parahippocampal gyrii, lateral occipital & intracalcarine cortices & hippocampus |
|  |  | 5.38 | -38 | -9 | 28 | **Left cerebral white matter** |  |
|  |  | 5.22 | 15 | -58 | 4 | **Right precuneus cortex** |  |
| <0.001  (Cluster 2) | 5807 | 4.52 | 0 | -62 | 56 | **Precuneus** |  |
|  |  | 4.36 | 3 | -56 | 60 | **Precuneus** |  |
|  |  | 3.97 | -3 | -30 | 34 | **Posterior cingulate** |  |
| <0.001  (Cluster 3) | 3343 | 4.25 | 62 | -15 | 4 | **Right planum temporale** | - Right supramarginal, middle frontal, inferior frontal, postcentral, superior temporal & Heschl's gyrii & posterior insula |
|  |  | 4.15 | 39 | -12 | 18 | **Right central oppercular cortex** |  |
|  |  | 3.95 | 36 | -4 | 32 | **Right cerebral white matter** |  |
| **Combined-Reward > Neutral anticipation: Fentanyl > Naloxone** | | | | | | | |
| Cluster-level p-FWE | Cluster size (vox) | Local maxima  t-stat | MNI coordinates (mm) | | | Cluster Regional Details | |
|  |  |  | X | Y | Z | Local Maxima | Other regions within cluster |
| 0.039  (Cluster 4) | 1039 | 4.76 | 28 | 32 | 45 | **Right middle frontal gyrus** | - Right superior frontal gyrus |
|  |  | 3.86 | 27 | 46 | 39 | **Right frontal pole** |  |
|  |  | 3.68 | 44 | 15 | 51 | **Right middle frontal gyrus** |  |
| 0.002  (Cluster 5) | 2355 | 4.66 | 2 | -26 | 33 | **Posterior cingulate** |  |
|  |  | 4.54 | -4 | -20 | 30 | **Posterior cingulate** |  |
|  |  | 4.34 | -4 | -8 | 30 | **Anterior cingulate** |  |
| 0.030  (Cluster 6) | 1136 | 4.54 | -39 | 27 | 44 | **Left middle frontal gyrus** | - Left superior frontal gyrus |
|  |  | 4.14 | -28 | 39 | 40 | **Left frontal pole** |  |
|  |  | 3.47 | -30 | 22 | 52 | **Left middle frontal gyrus** |  |
| <0.001  (Cluster 7) | 3166 | 4.43 | 9 | -48 | -10 | **Cerebellum** | - Bilateral lingual, lateral occipitotemporal fusiform & parahippocampal gyrii, hippocampus & occipital pole |
|  |  | 4.34 | -24 | -36 | -4 | **Left hippocampus** |  |
|  |  | 4.33 | 12 | -30 | -10 | **Brain stem** |  |
| 0.006  (Cluster 8) | 1758 | 4.42 | -9 | 51 | 4 | **Paracingulate gyrus** |  |
|  |  | 4.41 | 18 | 62 | -4 | **Right frontal pole** |  |
|  |  | 4.35 | 0 | 58 | 2 | **Frontal pole** |  |

| **Combined Loss > Neutral anticipation: Fentanyl > Placebo** | | | | | | | | |
| --- | --- | --- | --- | --- | --- | --- | --- | --- |
| Cluster-level p-FWE | Cluster size (vox) | Local maxima  t-stat | MNI coordinates (mm) | | | Cluster Regional Details | | |
|  |  |  | X | Y | Z | Local Maxima | Other regions within cluster | |
| 0.043  (Cluster 9) | 1085 | 4.05 | -26 | -63 | 9 | **Left intracalcarine cortex** | |  |
|  |  | 3.69 | -20 | -80 | 3 | **Left intracalcarine cortex** | |  |
|  |  | 3.68 | -9 | -69 | 0 | **Left lingual gyrus** | |  |
| **Combined Loss > Neutral anticipation: Fentanyl > Placebo** | | | | | | | | |
| Cluster-level p-FWE | Cluster size (vox) | Local maxima  t-stat | MNI coordinates (mm) | | | Cluster Regional Details | | |
|  |  |  | X | Y | Z | Local Maxima | | Other regions within cluster |
| <0.001  (Cluster 10) | 5634 | 6.79 | -36 | 26 | 42 | **Left middle frontal gyrus** | | - Bilateral superior frontal & paracingulate gyrii |
|  |  | 6.25 | 30 | 30 | 46 | **Right middle frontal gyrus** | |  |
|  |  | 5.53 | -27 | 20 | 38 | **Left middle frontal gyrus** | |  |
| 0.002 | 2481 | 4.87 | 16 | 62 | -6 | **Frontal pole** | | - Paracingulate gyrus |
| (Cluster 11) |  | 4.73 | -3 | 60 | -2 | **Frontal pole** | |  |
| 0.005  (Cluster 12) | 1987 | 4.57 | -14 | -48 | -8 | **Left lingual gyrus** | | - Left insula and temporal fusiform cortex |
|  |  | 4.25 | -6 | -58 | -6 | **Left lingual gyrus** | |  |
|  |  | 4.05 | -38 | -15 | -12 | **Left Cerebral White matter** | |  |
| 0.004  (Cluster 13) | 2143 | 4.06 | -3 | -36 | 42 | **Posterior cingulate** | | - Intracalcarine cortex |
|  |  | 4.00 | -2 | -60 | 20 | **Precuneous** | |  |
|  |  | 3.83 | 2 | -44 | 36 | **Posterior cingulate** | |  |

**Table S6** – Exploratory whole-brain analyses for other anticipation contrasts: significant results. Including cluster regional details from Harvard-Oxford Cortical and Subcortical Structural Atlases. Local cluster maxima locations in bold

| **High Reward > Neutral anticipation: Fentanyl > Placebo** | | | | | | | | | |
| --- | --- | --- | --- | --- | --- | --- | --- | --- | --- |
| Cluster-level p-FWE | Cluster size (vox) | | Local maxima  t-stat | MNI coordinates (mm) | | | Cluster Regional Details | | |
|  |  |  |  | X | Y | Z | Local Maxima | Other regions within cluster | |
| <0.001 | 20452 | | 5.17 | -6 | -50 | 0 | **Left lingual gyrus** | - Left supramarginal gyrus & superior parietal lobule  - Bilateral occipital & temporal fusiform, inferior temporal gyri and intracalcarine and lateral occipital cortices, hippocampus | |
|  |  |  | 4.91 | 14 | -56 | 6 | **Right lingual gyrus** |  |  |
|  |  |  | 4.85 | 8 | -48 | 2 | **Posterior cingulate** |  |  |
| <0.001 | 3678 | | 4.49 | 0 | -51 | 69 | **Precuneus** |  | |
|  |  |  | 4.23 | 15 | -46 | 50 | **Precuneus** |  |  |
|  |  |  | 4.20 | 0 | -62 | 56 | **Precuneus** |  |  |
| **High Reward > Neutral anticipation: Fentanyl > Naloxone** | | | | | | | | | |
| Cluster-level p-FWE | Cluster size (vox) | Local maxima  t-stat | | MNI coordinates (mm) | | | Cluster Regional Details | | |
|  |  |  |  | X | Y | Z | Local Maxima | | Other regions within cluster |
| 0.012 | 1512 | 4.96 | | -3 | 48 | 4 | **Paracingulate gyrus** | |  |
|  |  | 4.56 | | 20 | 60 | -6 | **Frontal pole** | |  |
|  |  | 4.12 | | 6 | 52 | 2 | **Paracingulate gyrus** | |  |

| **High Loss > Neutral anticipation: Fentanyl > Naloxone** | | | | | | | |
| --- | --- | --- | --- | --- | --- | --- | --- |
| Cluster-level p-FWE | Cluster size (vox) | Local maxima  t-stat | MNI coordinates (mm) | | | Cluster Regional Details | |
|  |  |  | X | Y | Z | Local Maxima | Other regions within cluster |
| 0.003 | 2297 | 6.2 | -36 | 26 | 42 | **Left middle frontal gyrus** |  |
|  |  | 4.24 | -22 | 33 | 46 | **Left superior frontal gyrus** |  |
|  |  | 3.74 | -46 | 21 | 26 | **Left middle frontal gyrus** |  |
| 0.005 | 2001 | 4.5 | -2 | -60 | 20 | **Precuneus** |  |
|  |  | 3.92 | -2 | -42 | 32 | **Posterior cingulate gyrus** |  |
|  |  | 3.79 | 6 | -48 | 21 | **Posterior cingulate gyrus** |  |
| 0.048 | 1029 | 4.35 | -2 | 52 | 4 | **Paracingulate gyrus** |  |
|  |  | 3.88 | 16 | 62 | -6 | **Frontal pole** |  |
|  |  | 3.81 | 9 | 60 | -9 | **Frontal pole** |  |
| **Low Reward > Neutral anticipation: Fentanyl > Placebo** | | | | | | | |
| Cluster-level p-FWE | Cluster size (vox) | Local maxima  t-stat | MNI coordinates (mm) | | | Cluster Regional Details | |
|  |  |  | X | Y | Z | Local Maxima | Other regions within cluster |
| <0.001 | 10994 | 4.99 | 24 | -45 | -8 | **Right lingual gyrus** | - Right precuneous, occipital fusiform & parahippocampal gyri, thalamus, hippocampus  - Bilateral posterior cingulate, occipital pole, intracalcarine cortex |
|  |  | 4.83 | 14 | -26 | -12 | **Brain stem** |  |
|  |  | 4.61 | 14 | -33 | -22 | **Brain stem** |  |
| 0.023 | 1119 | 4.08 | -12 | -40 | 44 | **Precuneus** |  |
|  |  | 3.92 | -4 | -33 | 34 | **Posterior cingulate** |  |
|  |  | 3.71 | -10 | -36 | 28 | **Left cerebral white matter** |  |
| 0.045 | 904 | 4.03 | 2 | -60 | 56 | **Precuneus** |  |
|  |  | 4.01 | 6 | -54 | 60 | **Precuneus** |  |
|  |  | 3.69 | -4 | -75 | 46 | **Precuneus** |  |
|  |  | 4.01 | 6 | -54 | 60 | **Precuneus** |  |
|  |  | 3.69 | -4 | -75 | 46 | **Precuneus** |  |
| **Low Reward > Neutral anticipation: Fentanyl > Naloxone** | | | | | | | |
| Cluster-level p-FWE | Cluster size (vox) | Local maxima  t-stat | MNI coordinates (mm) | | | Cluster Regional Details | |
|  |  |  | X | Y | Z | Local Maxima | Other regions within cluster |
| 0.016 | 1239 | 4.29 | 9 | -4 | 30 | **Right cerebral white matter** |  |
|  |  | 4.28 | -6 | -6 | 30 | **Anterior cingulate** |  |
|  |  | 3.87 | -6 | -20 | 30 | **Posterior cingulate** |  |

| **Low Loss > Neutral anticipation: Fentanyl > Naloxone** | | | | | | | |
| --- | --- | --- | --- | --- | --- | --- | --- |
| Cluster-level p-FWE | Cluster size (vox) | Local maxima  t-stat | MNI coordinates (mm) | | | Cluster Regional Details |  |
|  |  |  | X | Y | Z |  |  |
| <0.001 | 2857 | 4.98 | -32 | 24 | 45 | **Left middle frontal gyrus** | Left paracingulate gyrus & frontal pole |
|  |  | 4.78 | -27 | 32 | 36 | **Left middle frontal gyrus** |  |
|  |  | 4.36 | -26 | 22 | 54 | **Left superior frontal gyrus** |  |
| 0.002 | 2178 | 4.9 | 18 | 58 | -6 | **Frontal pole** |  |
|  |  | 3.98 | 3 | 50 | -6 | **Paracingulate gyrus** |  |
|  |  | 3.96 | -10 | 42 | 15 | **Paracingulate gyrus** |  |
| 0.030 | 1082 | 4.73 | -14 | -48 | -8 | **Left lingual gyrus** | Left hippocampus |
|  |  | 3.6 | -12 | -36 | -9 | **Left parahippocampal gyrus** |  |
|  |  | 3.48 | -9 | -62 | -2 | **Left lingual gyrus** |  |
| 0.006 | 1673 | 4.64 | -39 | 44 | -4 | **Left frontal pole** | Left insula cortex |
|  |  | 4.54 | -39 | -15 | -14 | **Left cerebral white matter** |  |
|  |  | 4.11 | -36 | 20 | -12 | **Left orbitofrontal cortex** |  |

**Table S7** – Whole-brain analyses examining fentanyl-1 and fentanyl-2 vs placebo and naloxone for combined-reward and combined-loss anticipation contrasts: significant results. Including local cluster maxima locations in bold from Harvard-Oxford Cortical and Subcortical Structural Atlases

| **Combined-Reward > Neutral anticipation: Fentanyl-1 > Placebo** | | | | | | | | | |
| --- | --- | --- | --- | --- | --- | --- | --- | --- | --- |
| Cluster-level p-FWE | Cluster size (vox) | | Local maxima  t-stat | MNI coordinates (mm) | | | Cluster Regional Details | | |
|  |  |  |  | X | Y | Z | Local Maxima |  | |
| 0.046 | 935 | | 3.83 | 8 | -50 | 2 | **Right Lingual Gyrus / Posterior Cingulate** |  | |
|  |  |  | 3.71 | 15 | -52 | 8 | **Right Precuneus** |  |  |
|  |  |  | 3.50 | -4 | -46 | -3 | **4^th^ Ventricle** |  |  |
| **Combined-Reward > Neutral anticipation: Fentanyl-2 > Placebo** | | | | | | | | | |
| Cluster-level p-FWE | Cluster size (vox) | Local maxima  t-stat | | MNI coordinates (mm) | | | Cluster Regional Details | | |
|  |  |  |  | X | Y | Z | Local Maxima | |  |
| <0.001 | 45725 | 5.50 | | 15 | -58 | 4 | **Right Lingual Gyrus** | |  |
|  |  | 5.44 | | 9 | -48 | 0 | **Precuneus** | |  |
|  |  | 5.18 | | 14 | -60 | -8 | **Right Lingual Gyrus** | |  |
| 0.001 | 2504 | 4.61 | | 64 | -16 | 9 | **Right Planum Temporale** | |  |
|  |  | 4.60 | | 39 | -15 | 15 | **Right Insular Cortex** | |  |
|  |  | 4.53 | | 62 | -2 | 9 | **Right Central Opercular Cortex** | |  |
| 0.049 | 915 | 4.04 | | 33 | 21 | 18 | **Right Frontal Operculum Cortex** | |  |
|  |  | 3.96 | | 42 | 22 | 20 | **Right Inferior Frontal Gyrus** | |  |
|  |  | 3.63 | | 27 | 28 | 36 | **Right Middle Frontal Gyrus** | |  |

| **Combined-Reward > Neutral anticipation: Fentanyl-2 > Naloxone** | | | | | | | |
| --- | --- | --- | --- | --- | --- | --- | --- |
| Cluster-level p-FWE | Cluster size (vox) | Local maxima  t-stat | MNI coordinates (mm) | | | Cluster Regional Details | |
|  |  |  | X | Y | Z | Local Maxima |  |
| <0.001 | 5279 | 5.53 | -4 | -20 | 30 | **Posterior Cingulate** |  |
|  |  | 4.79 | 8 | -6 | 30 | **Anterior Cingulate** |  |
|  |  | 4.58 | -6 | 9 | 24 | **Anterior Cingulate** |  |
| <0.001 | 6846 | 5.38 | -9 | 50 | 3 | **Left Paracingulate Gyrus** |  |
|  |  | 5.25 | -40 | 27 | 44 | **Left Middle Frontal Gyrus** |  |
|  |  | 5.22 | -40 | 24 | 34 | **Left Middle Frontal Gyrus** |  |
| <0.001 | 7794 | 4.79 | 8 | -94 | 2 | **Occipital Pole** |  |
|  |  | 4.77 | 9 | -50 | -12 | **Right Lingual Gyrus** |  |
|  |  | 4.61 | -24 | -38 | -4 | **Left Hippocampus** |  |
| **Combined-Loss > Neutral anticipation: Fentanyl-1 > Naloxone** | | | | | | | |
| Cluster-level p-FWE | Cluster size (vox) | Local maxima  t-stat | MNI coordinates (mm) | | | Cluster Regional Details | |
|  |  |  | X | Y | Z | Local Maxima |  |
| 0.012 | 1601 | 4.93 | -34 | 24 | 44 | **Left Middle Frontal Gyrus** |  |
|  |  | 4.51 | -27 | 18 | 36 | **Left Middle Frontal Gyrus** |  |
|  |  | 3.64 | -27 | 32 | 38 | **Left Middle Frontal Gyrus** |  |
| **Combined-Loss > Neutral anticipation: Fentanyl-2 > Naloxone** | | | | | | | |
| Cluster-level p-FWE | Cluster size (vox) | Local maxima  t-stat | MNI coordinates (mm) | | | Cluster Regional Details | |
|  |  |  | X | Y | Z | Local Maxima |  |
| <0.001 | 5906 | 6.52 | -36 | 26 | 42 | **Left Middle Frontal Gyrus** |  |
|  |  | 5.68 | 28 | 32 | 45 | **Right Middle Frontal Gyrus** |  |
|  |  | 5.20 | -30 | 22 | 38 | **Left Middle Frontal Gyrus** |  |
| 0.06 | 958 | 4.83 | 57 | -63 | 18 | **Right Lateral Occipital Cortex** |  |
|  |  | 4.57 | 54 | -57 | 14 | **Right Anglar Gyrus** |  |
|  |  | 4.35 | 51 | -68 | 26 | **Right Lateral Occipital Cortex** |  |
| <0.001 | 3834 | 4.75 | 38 | 46 | -6 | **Right Frontal Pole** |  |
|  |  | 4.75 | -3 | 62 | -3 | **Frontal Pole** |  |
|  |  | 4.60 | 18 | 62 | -4 | **Right Frontal Pole** |  |
| 0.004 | 2170 | 4.33 | -3 | -56 | 32 | **Precuneus** |  |
|  |  | 4.28 | 0 | -60 | 20 | **Precuneus** |  |
|  |  | 3.90 | 12 | -54 | 15 | **Precuneus** |  |

**Table S8** – End tidal CO_2_ and respiratory rate

|  | **Fentanyl-1** | **Fentanyl-2** | **Naloxone** | **Placebo** | **Repeated measure ANOVA  p-value** |
| --- | --- | --- | --- | --- | --- |
| Mean MID task EtCO2 (mmHg) | 40.5  (±2.7) | 40.5 (±3.9) | 38.9* (1.4) | 38.7*†  (1.5) | 0.044 |
| Change in mean EtCO2 compared with placebo (mmHg) | +1.6 | +1.6 | +0.2 | N/A |  |
| ∆EtCO2 (pre- vs. post-drug infusion) | 0.96  (±0.04) | 0.97 (±0.06) | 1.00* (±0.03) | 1.00*† (±0.02) | <0.001 |
| Mean MID task respiratory rate  (breaths/min) | 14.8 (±2.6) | 14.4 (±1.9) | 15.8† (±2.2) | 16.1*† (±2.4) | 0.007 |

*Paired sample t-test:
* compared with Fentanyl-1, p<0.05*

*† compared with Fentanyl-2, p<0.05
all other p>0.05*

**Figure S2** – Comparison of significant clusters in n=14 participants with EtCO2 data for whole-brain models including and excluding ∆EtCO2 as a covariate in the model. Presented in neurological orientation (i.e. image left is subject’s left).

Total equivalent sizes of significant cluster (K_E_) for:
A) Excluding EtCO_2_ K_E_=20122, including EtCO_2_ K_E_=1774
B) Excluding EtCO_2_ K_E_=4145, including EtCO_2_ K_E_=2347

**Table S9** – [^11^C]-carfentanil BP_ND_ for Harvard Oxford cortical and sub-cortical atlas ROIs and significant combined-reward>neutral and combined-loss>neutral anticipation clusters from whole brain analyses. Regions in BOLD ROIs included in significant clusters in our a priori whole brain analyses.

| **Rank** | **Harvard Oxford Region** | **[^11^C]-carfentanil BP_ND_** | **Result Cluster** |
| --- | --- | --- | --- |
| 1 | Nucleus accumbens (ventral striatum) | 2.79 |  |
| 2 | Thalamus | 1.78 |  |
| 3 | Putamen | 1.55 |  |
| 4 | Caudate | 1.45 |  |
| 5 | Amygdala | 1.44 |  |
| 6 | **Paracingulate gyrus** | 1.28 |  |
| 7 | **Anterior cingulate gyrus** | 1.26 |  |
| 8 | Subcallosal cortex | 1.22 |  |
| 9 | **Insular cortex** | 1.21 |  |
| 10 | Frontal medial cortex | 1.20 |  |
| 11 |  | 1.16 | **Combined-Reward > Neutral anticipation**  Fentanyl > Naloxone (Cluster 4) *- Right Middle frontal gyrus and frontal pole* |
| 12 | Frontal operculum cortex | 1.15 |  |
| 13 |  | 1.14 | **Combined-Reward > Neutral anticipation**  Fentanyl > Naloxone (Cluster 6) *- Left* *Middle frontal gyrus and frontal pole* |
| 14 |  | 1.08 | **Combined-Loss > Neutral anticipation**  Fentanyl > Naloxone (Cluster 11)  *- Frontal pole, left lingual gyrus* |
| 15 | Frontal orbital cortex | 1.05 |  |
| 16 |  | 1.05 | **Combined-Reward > Neutral anticipation** Fentanyl > Naloxone (Cluster 8)  *- paracingulate gyrus, frontal pole* |
| 17 |  | 1.02 | **Combined-Loss > Neutral anticipation**  Fentanyl > Naloxone (Cluster 10)  *- Bilateral middle frontal gyrus* |
| 18 | Inferior temporal gyrus, anterior division | 1.02 |  |
| 19 | **Temporal fusiform cortex, anterior division** | 1.00 |  |
| 20 | **Central opercular cortex** | 0.97 |  |
| 21 | **Inferior frontal gyrus, pars opercularis** | 0.96 |  |
| 22 | Inferior temporal gyrus, posterior division | 0.96 |  |
| 23 | Juxtapositional lobule cortex | 0.95 |  |
| 24 | Middle temporal gyrus, anterior division | 0.93 |  |
| 25 | **Superior frontal gyrus** | 0.93 |  |
| 26 | Planum polare | 0.91 |  |
| 27 |  | 0.91 | **Combined-Loss > Neutral anticipation** Fentanyl > Naloxone (Cluster 12)  *- Left lingual gyrus* |
| 28 | **Middle frontal gyrus** | 0.91 |  |
| 29 | **Superior temporal gyrus, anterior division** | 0.91 |  |
| 30 | Temporal pole | 0.88 |  |
| 31 | Middle temporal gyrus, posterior division | 0.88 |  |
| 32 | **Supramarginal gyrus, anterior division** | 0.87 |  |
| 33 | Parietal operculum cortex | 0.86 |  |
| 34 | **Frontal pole** | 0.86 |  |
| 35 | **Inferior frontal gyrus, pars triangularis** | 0.86 |  |
| 36 | Angular gyrus | 0.84 |  |
| 37 |  | 0.84 | **Combined-Reward > Neutral anticipation** Fentanyl > Placebo (Cluster 2)  *- Precuneus, posterior cingulate* |
| 38 | **Supramarginal gyrus, posterior division** | 0.84 |  |
| 39 | **Temporal fusiform cortex, posterior division** | 0.84 |  |
| 40 | Middle temporal gyrus, temporooccipital part | 0.81 |  |
| 41 | Inferior temporal gyrus, temporooccipital part | 0.81 |  |
| 42 | **Posterior cingulate gyrus** | 0.80 |  |
| 43 | **Superior temporal gyrus, posterior division** | 0.72 |  |
| 44 | Pallidum | 0.70 |  |
| 45 |  | 0.70 | ***Combined-Reward > Neutral anticipation***  *Fentanyl >* Naloxone (Cluster 5)  *- Posterior and anterior cingulate* |
| 46 |  | 0.68 | **Combined-Reward > Neutral anticipation** Fentanyl > Placebo (Cluster3)  *- right planum temporale, oppercular cortex* |
| 47 | **Parahippocampal gyrus, anterior division** | 0.68 |  |
| 48 | **Temporal occipital fusiform cortex** | 0.67 |  |
| 49 | Superior parietal lobule | 0.64 |  |
| 50 | **Planum temporale** | 0.64 |  |
| 51 | **Precuneus cortex** | 0.63 |  |
| 52 | **Precentral gyrus** | 0.60 |  |
| 53 | **Heschls gyrus** | 0.56 |  |
| 54 | **Lateral occipital cortex, superior division** | 0.50 |  |
| 55 | **Hippocampus** | 0.48 |  |
| 56 | **Parahippocampal gyrus, posterior division** | 0.47 |  |
| 57 | **Postcentral gyrus** | 0.46 |  |
| 58 |  | 0.46 | **Combined-Loss > Neutral anticipation**  Fentanyl > Naloxone (Cluster 12)  *- Left lingual gyrus* |
| 59 | **Lateral occipital cortex, inferior division** | 0.41 |  |
| 60 |  | 0.38 | **Combined-Reward > Neutral anticipation**  Fentanyl > naloxone (Cluster 7)  - *Cerebellum, left hippocampus* |
| 61 |  | 0.33 | **Combined-Reward > Neutral anticipation** Fentanyl > Placebo (Cluster 1)  *- Right lingual gyrus, precuneus* |
| 62 | Occipital fusiform gyrus | 0.24 |  |
| 63 | **Lingual gyrus** | 0.17 |  |
| 64 |  | 0.15 | **Combined-Loss > Neutral anticipation**  Fentanyl > Placebo (Cluster 9)  *- Left intracalcarine cortex, lingual gyrus* |
| 65 | Cuneal cortex | 0.13 |  |
| 66 | Supracalcarine cortex | 0.12 |  |
| 67 | **Intracalcarine cortex** | 0.03 |  |
| 68 | **Occipital pole** | 0.02 |  |

**SUPPLEMENTARY REFERENCES**

Abler, B., Erk, S., & Walter, H. (2007). Human reward system activation is modulated by a single dose of olanzapine in healthy subjects in an event-related, double-blind, placebo-controlled fMRI study. *Psychopharmacology*, *191*(3), 823–833. https://doi.org/10.1007/s00213-006-0690-y

Ashburner, J. (2007). A fast diffeomorphic image registration algorithm. *NeuroImage*, *38*(1), 95–113. https://doi.org/10.1016/j.neuroimage.2007.07.007

Burt, J. B., Helmer, M., Shinn, M., Anticevic, A., & Murray, J. D. (2020). Generative modeling of brain maps with spatial autocorrelation. *NeuroImage*, *220*, 117038. https://doi.org/10.1016/j.neuroimage.2020.117038

Cammoun, L., Gigandet, X., Meskaldji, D., Thiran, J. P., Sporns, O., Do, K. Q., Maeder, P., Meuli, R., & Hagmann, P. (2012). Mapping the human connectome at multiple scales with diffusion spectrum MRI. *Journal of Neuroscience Methods*, *203*(2), 386–397. https://doi.org/10.1016/j.jneumeth.2011.09.031

Chen, Y., Chaudhary, S., & Li, C.-S. R. (2022). Shared and distinct neural activity during anticipation and outcome of win and loss: A meta-analysis of the monetary incentive delay task. *NeuroImage*, *264*, 119764. https://doi.org/10.1016/j.neuroimage.2022.119764

First, M. B., Spitzer, R. L., Gibbon, M., & Williams, J. (2002). *Structured clinical interview for DSM-IV-TR Axis I disorders, research version*. Biometrics Research, New York State Psychiatric Institute.

Kantonen, T., Karjalainen, T., Isojärvi, J., Nuutila, P., Tuisku, J., Rinne, J., Hietala, J., Kaasinen, V., Kalliokoski, K., Scheinin, H., Hirvonen, J., Vehtari, A., & Nummenmaa, L. (2020). Interindividual variability and lateralization of μ-opioid receptors in the human brain. *NeuroImage*, *217*, 116922. https://doi.org/10.1016/J.NEUROIMAGE.2020.116922

Oldham, S., Murawski, C., Fornito, A., Youssef, G., Yücel, M., & Lorenzetti, V. (2018). The anticipation and outcome phases of reward and loss processing: A neuroimaging meta‐analysis of the monetary incentive delay task. *Human Brain Mapping*, *39*(8), 3398–3418. https://doi.org/10.1002/hbm.24184

Power, J. D., Barnes, K. A., Snyder, A. Z., Schlaggar, B. L., & Petersen, S. E. (2012). Spurious but systematic correlations in functional connectivity MRI networks arise from subject motion. *NeuroImage*, *59*(3), 2142–2154. https://doi.org/10.1016/j.neuroimage.2011.10.018

Siegel, J. S., Power, J. D., Dubis, J. W., Vogel, A. C., Church, J. A., Schlaggar, B. L., & Petersen, S. E. (2014). Statistical improvements in functional magnetic resonance imaging analyses produced by censoring high-motion data points: Censoring High Motion Data in fMRI. *Human Brain Mapping*, *35*(5), 1981–1996. https://doi.org/10.1002/hbm.22307

Virtanen, P., Gommers, R., Oliphant, T. E., Haberland, M., Reddy, T., Cournapeau, D., Burovski, E., Peterson, P., Weckesser, W., Bright, J., Van Der Walt, S. J., Brett, M., Wilson, J., Millman, K. J., Mayorov, N., Nelson, A. R. J., Jones, E., Kern, R., Larson, E., … Vázquez-Baeza, Y. (2020). SciPy 1.0: Fundamental algorithms for scientific computing in Python. *Nature Methods*, *17*(3), 261–272. https://doi.org/10.1038/s41592-019-0686-2

Wilson, R. P., Colizzi, M., Bossong, M. G., Allen, P., Kempton, M., Bhattacharyya, S., & Bhattacharyya, S. (2018). The Neural Substrate of Reward Anticipation in Health: A Meta-Analysis of fMRI Findings in the Monetary Incentive Delay Task. *Neuropsychology Review*, *28*(4), 496–506. https://doi.org/10.1007/s11065-018-9385-5

Zelaya, F. O., Zois, E., Muller-Pollard, C., Lythgoe, D. J., Lee, S., Andrews, C., Smart, T., Conrod, P., Vennart, W., Williams, S. C. R., Mehta, M. A., & Reed, L. J. (2012). The response to rapid infusion of fentanyl in the human brain measured using pulsed arterial spin labelling. *Magnetic Resonance Materials in Physics, Biology and Medicine*, *25*(2), 163–175. https://doi.org/10.1007/s10334-011-0293-4
